# Genetic Rearrangements Can Modify Chromatin Features at Epialleles

**DOI:** 10.1371/journal.pgen.1002331

**Published:** 2011-10-20

**Authors:** Andrea M. Foerster, Huy Q. Dinh, Laura Sedman, Bonnie Wohlrab, Ortrun Mittelsten Scheid

**Affiliations:** 1 Gregor Mendel Institute of Molecular Plant Biology (GMI), Austrian Academy of Sciences, Vienna, Austria; 2 Center for Integrative Bioinformatics Vienna, Max F. Perutz Laboratories (MFPL), Vienna, Austria; National Institute of Genetics, Japan

## Abstract

Analogous to genetically distinct alleles, epialleles represent heritable states of different gene expression from sequence-identical genes. Alleles and epialleles both contribute to phenotypic heterogeneity. While alleles originate from mutation and recombination, the source of epialleles is less well understood. We analyze active and inactive epialleles that were found at a transgenic insert with a selectable marker gene in *Arabidopsis*. Both converse expression states are stably transmitted to progeny. The silent epiallele was previously shown to change its state upon loss-of-function of *trans*-acting regulators and drug treatments. We analyzed the composition of the epialleles, their chromatin features, their nuclear localization, transcripts, and homologous small RNA. After mutagenesis by T-DNA transformation of plants carrying the silent epiallele, we found new active alleles. These switches were associated with different, larger or smaller, and non-overlapping deletions or rearrangements in the 3′ regions of the epiallele. These *cis*-mutations caused different degrees of gene expression stability depending on the nature of the sequence alteration, the consequences for transcription and transcripts, and the resulting chromatin organization upstream. This illustrates a tight dependence of epigenetic regulation on local structures and indicates that sequence alterations can cause epigenetic changes at some distance in regions not directly affected by the mutation. Similar effects may also be involved in gene expression and chromatin changes in the vicinity of transposon insertions or excisions, recombination events, or DNA repair processes and could contribute to the origin of new epialleles.

## Author Summary

In contrast to alleles, epialleles have identical DNA sequence and differ only in gene expression and chromatin features. Epialleles are heritable and can also contribute to phenotypes. How this variation originates is unclear. In this study, we analyzed two epialleles found in *Arabidopsis* for the difference between their chromatin features and their potential to change state. We mutagenized plants with the inactive epiallele and recovered mutants with restored gene expression. In several cases, this was connected with different rearrangements downstream of the epiallele that caused a switch of the epigenetic configuration further upstream. Therefore, sequence alterations, for example by transposon activity or recombination events, may trigger similar heritable changes of chromatin and gene expression in their proximity and could create new epialleles.

## Introduction

Epialleles are heritable states of different gene expression from sequence-identical genes and have been described in several organisms [1]–[3]. Like genetically different alleles, epialleles contribute to phenotypic heterogeneity [4]–[5]. While the mutagenic processes creating DNA sequence allele variations are relatively well understood, little is known about how and when epialleles originate, and it is difficult to investigate this *in statu nascendi*. In plants, epialleles were described as natural variants [6]–[9], mutation-induced [10]–[12], or associated with tissue-culture [13]–[15]. Once established, epialleles can acquire stability over many generations; however, they have much higher reversion rates than genetic alleles. Therefore, analyzing the switch from one epigenetic state to the other at well-characterized epialleles can provide insight into their natural origin.

Pairs of epialleles are characterized by antithetic histone modifications at the associated nucleosomes, transcriptional activity at the expressed form, and transcriptional gene silencing (TGS) at the other. In some fungi, mammals, and higher plants, the latter is connected with cytosine methylation at the epiallele [e.g. 6], [16]–[17]. Several pairs of epialleles in plants define easily scored phenotypes like morphology [6], [10], development [9], pigmentation [7], [18], or reporter gene expression [19]–[20]. Some epialleles, as well as many other epigenetically controlled genes, have been used for mutant screens and have helped identify many different proteins and RNAs whose presence or absence can cause transient or stable changes of epiallele expression, or influence epigenetic regulation in general. There is also a wealth of data on the influence of drug treatments, sequence determinants, and the role of genomic neighborhood, on epigenetic regulation.

*Arabidopsis thaliana* has been the plant model of choice for genetic analysis of switching between epiallelic states, based on the rich genetic and genomic resources available. The experimental system in our study is based on a pair of epialleles in *Arabidopsis thaliana* containing either an expressed or silent hygromycin phosphotransferase gene (*HPT*). Active transcription confers resistance to the antibiotic while the inactive epiallele renders the plant sensitive. Gene expression can be selected for on antibiotic-containing medium but does not affect the plants during non-selective growth. The epialleles were found in tetraploid plants obtained by regeneration from protoplasts [20]. While some lines had resistant progeny and expressed the *HPT* gene, other lines had silenced the *HPT* and produced only sensitive progeny. The R and S epialleles (determining resistance and sensitivity on hygromycin, respectively) were maintained in their particular expression state after diploidization and for all generations of self-pollination analyzed so far (Figure S1). Beside their differences in transcription, they also differ in DNA methylation [21]. We screened for a switch between the epialleles, by scoring for restored hygromycin resistance after T-DNA mutagenesis of the diploid S line. We identified two *trans*-acting factors whose nature indicated an epigenetic ‘double lock’ at the silent epiallele [22]. In contrast to many other silent genes, silencing could only be released by simultaneous interference with methylation of DNA and histones. Six mutations from the same screen were mapped to the resistance gene itself. These *cis*-mutations provided the opportunity to study the nature and effect of DNA sequence changes on gene expression, chromatin organization, and genetic stability. We describe these new alleles in detail and compare them with the R and S epialleles. We show that different, and non-overlapping, sequence changes downstream of the *HPT* gene can restore the expression of the upstream promoter, to a similar extent as the mutations interfering with the chromatin factors in *trans*. Such small sequence alterations that cause epigenetic changes at some distance may also be involved in gene expression and chromatin changes in the vicinity of transposon insertions/excisions, recombination events, or DNA repair processes and may thereby contribute to the origin of new epialleles.

## Results

### Epialleles Differ in Chromatin Features and Small RNA Abundance

**Figure 1:**
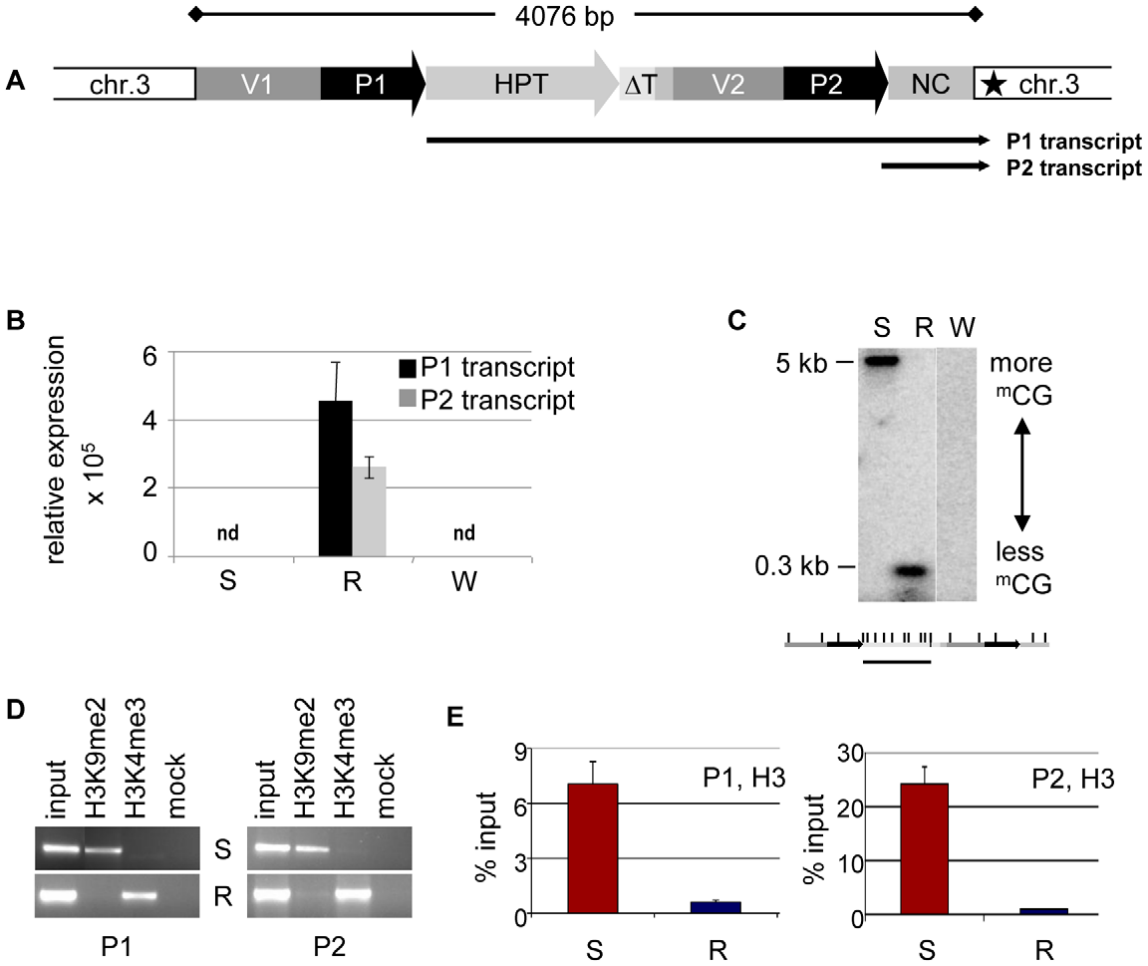
Epialleles differ in chromatin features. (A) Transgenic insert (identical in lines S and R) with duplicated vector (V1, V2) and CaMV35S promoter (P1, P2) sequences and single copies of the hygromycin phosphotransferase resistance gene (HPT, P1 transcript), a truncated terminator (ΔT) and a non-coding sequence containing bovine carrier DNA (NC, P2 transcript). Black star: polyadenylation signal-like sequence. (B) Transcript levels determined by qRT-PCR in diploid *Arabidopsis* ecotype Zürich with (S, R) or without (W, wild type) the transgenic insert. P1 was determined with primers within the HPT sequence, P2 with primers within the NC sequence. Due to the overlap, this might capture also some P1 templates. Normalization to S; reference gene *EIF4a* (At3g13920); error bars represent standard deviation of triplicate measurements. (C) Methylation analysis in three week-old seedlings with (S, R) or without (W, wild type) the transgenic insert. Genomic DNA was treated with *Hpa*II not cutting ^m^C^m^CGG, blotted and hybridized to a probe spanning the HPT sequence. Enzyme recognition sites are indicated below the blot. (D, E) Analysis of histone H3 at both promoters (P1, amplicon 133 bp; P2, amplicon 197 bp; primers see Table S4) in lines S and R by chromatin immunoprecipitation. (D) Association with H3K9me2 and H3K4me3; (E) modification-independent precipitation.

The *HPT* gene is inserted in an AT-rich intergenic region on *Arabidopsis thaliana* chromosome 3 [20]. Previous investigations, and published data from genome-wide screens for chromatin features [20], [23]–[24], indicated that the genomic localization itself is unlikely to influence the epigenetic state of the *HPT* gene, as no prominent epigenetic modifications are present in the neighborhood of the insertion. Resistant and sensitive *Arabidopsis* lines with the different epialleles had been generated from the same progenitor line homozygous for the *HPT* gene, thereby being supposedly isogenic. Nevertheless, the lack of transcription initiation in the hygromycin-sensitive lines could have been due to a DNA sequence mutation in a regulatory region, for example, a transcription factor binding site. Also, the structure of the insert had not been analyzed in detail. Therefore, active and inactive versions were amplified from genomic DNA of the respective lines. Both epialleles are potentially fully functional and have identical sequences. The 35S promoter (P1) is flanked upstream by a 661 bp fragment derived from the plasmid vector (V1). A rearrangement between two vector molecules prior to, or during, the integration of the transgene into the plant genome caused a duplication of the adjacent vector sequence (V2) and the 35S promoter (P2), resulting in two tandem repeats (Figure 1A). The polyadenylation signal from the CaMV 35S terminator following the *HPT* ORF lacks 151 bp compared to the transformation construct and has therefore lost its termination function (ΔT), causing read through of the P1 transcript into the flanking plant genome sequence (Figure 1A). P2 is followed by a 505 bp non-protein coding fragment (NC) harboring sequences of bovine carrier DNA used to assist PEG-mediated direct gene transfer to mesophyll protoplasts [25], interspersed with 54 nucleotides without homology to known sequences. This heterologous DNA is transcribed by P2, giving rise to a smaller non-coding transcript (P2 transcript) (Figure 1A). Resistant plants produce the longer P1 and the shorter P2 transcripts, while both promoters are inactive in sensitive plants (Figure 1B and Figure S6). Therefore, the isogenic inserts differ only by gene expression, and R and S represent true epialleles.

The different expression states were suspected to originate from distinct chromatin configuration, and previous studies had provided evidence for opposing DNA methylation at the epialleles, especially pronounced at the transcription factor binding sites ([20]–[21], Figure 1C). As DNA methylation and silencing are usually correlated with specific changes of the DNA-associated proteins, we investigated histone modifications and nucleosome occupancy at the epialleles by chromatin immunoprecipitation. This revealed significant differences between the epialleles along the whole transgenic insert. While expressing lines (R) were primarily marked by trimethylation of histone H3 at lysine residue 4 (H3K4me3), typically enriched in euchromatic regions, epialleles in silenced lines (S) have nucleosomes with a modification characteristic of heterochromatin, namely dimethylated lysines at position 9 (H3K9me2) (Figure 1D). These marks, also including low levels of H3 dimethylated at position 27 (H3K27me2), only extend a short distance from the transgene into the flanking plant DNA (Figure S2), indicating limited spreading in transcriptional direction. Beside the specific modifications, we also observed an overall reduced association with H3 in line R compared to S (Figure 1E), probably rendering the promoters more accessible for the transcription machinery. While the epialleles clearly differed in their local chromatin configuration, this did not have any effect on their nuclear localization (Figure S3).

Both epialleles were stably inherited over a minimum of eight generations of self-pollination, without any evidence for spontaneous switches in the germ line. To also study the stability of epialleles in undifferentiated cells, we initiated callus cultures, starting with cotyledons of resistant, sensitive, and non-transgenic plants, and propagated the calli for up to six months under non-selective conditions. We screened callus tissue at several time points for its ability to grow under hygromycin selection for up to 5 weeks. Calli derived from R lines were resistant whereas calli obtained from S or non-transgenic lines died on selection plates. We also determined chromatin modifications and DNA methylation in callus tissue grown on non-selective medium, with results comparable to those of leaf tissue (Figure S4). This demonstrates similar states and stable maintenance of epialleles even upon dedifferentiation.

**Figure 2:**
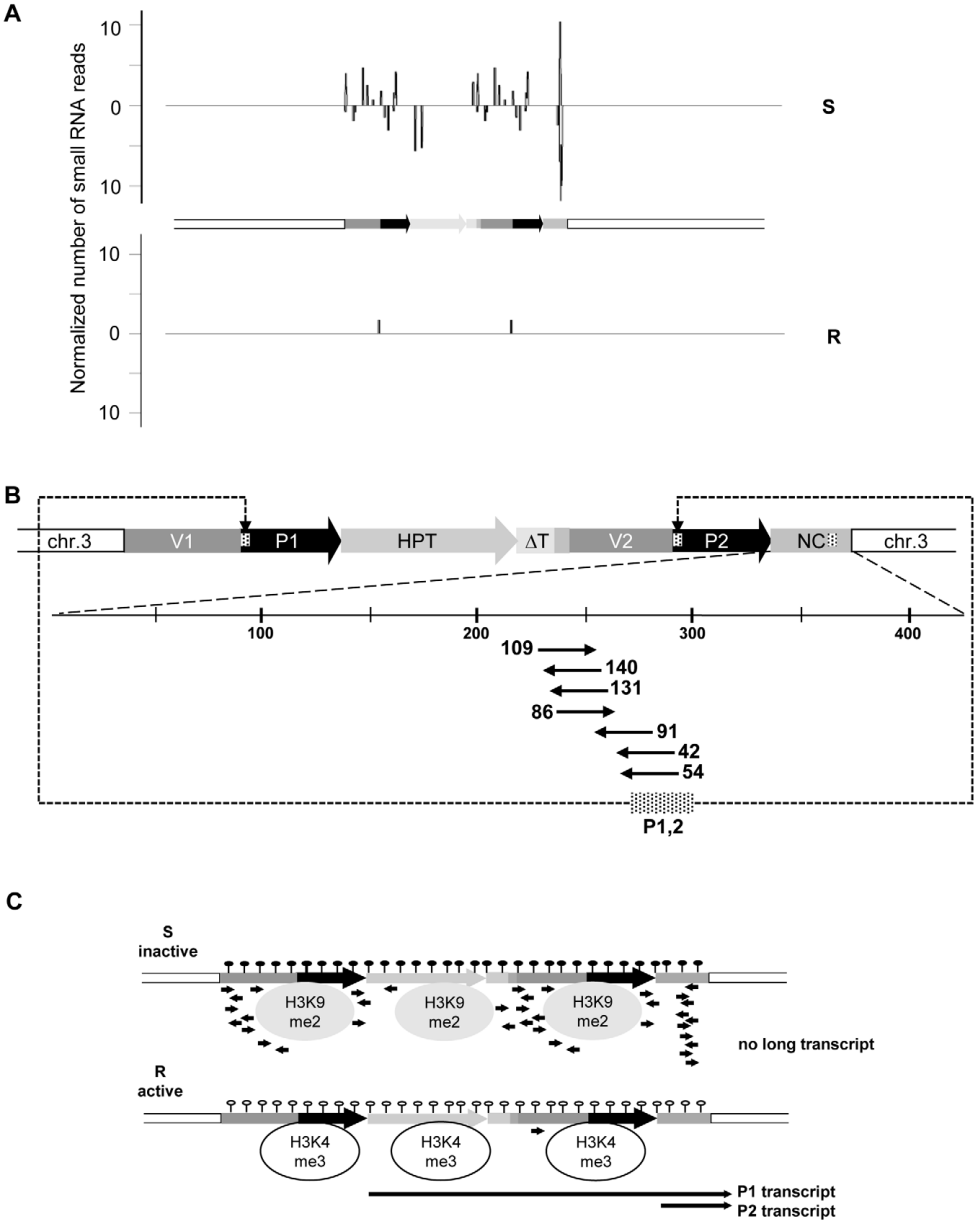
Epialleles differ in small RNA reads. (A) Abundance and location of small RNAs with homology to the inactive (S) and active (R) epiallele. Sense and antisense orientation are indicated above and below the horizontal lines, respectively. (B) Detailed view on the P2 transcript region and number of specific reads per million reads. Dashed region in P1, P2 and NC: position and overlap of reads with the promoter region. (C) Scheme of chromatin organization and RNA abundance at the inactive (S) and the active (R) epiallele. Filled and empty lollipops: presence or absence of DNA methylation; H3K9me2 and H3K4me3: modifications typical for transcriptionally silent and active chromatin, respectively; black arrows: RNA.

We screened for the involvement of antisense and/or small RNAs in silencing maintenance. Significant promoter activity of the NC region was excluded (Figure S5A), and specific antisense RNA in line S could also not be detected, neither by northern blotting (Figure S5B) nor by RT-PCR (data not shown). Nevertheless, we generated libraries from size-fractionated 19 nt to 26 nt RNAs prepared from flower buds of plants containing either the sensitive or resistant epiallele. Both libraries were sequenced (Table S1) and the reads screened for alignment with the transgenic insert. The library from the R plants had only 59 reads (3 per 1 million reads) with only one sequence with a match in the epiallele (Figure 2A, Table S3). In line S, we found 2661 (129 per 1 million reads) matching the epiallele, with a predominant length of 24 nucleotides (Figure 2A, Table S2 and Table S3), the size class known to be primarily involved in RNA-directed DNA methylation (RdDM). This is significantly more than in R, but still relatively little, compared to an individual miRNA (820 reads per 1 million for miRNA165) or to siRNA from a repetitive sequence (>1000 reads per 1 million for TSI [26]). The reads in S were distributed along the epiallele but mostly outside the *HPT* coding region. Importantly, among all reads specific for the silent epiallele we found an sRNA peak (671 reads, 476 antisense and 195 sense) covering 61 bp in the middle of the 505 bp non-coding sequence of the P2 transcript (Figure 2B). The most abundant sRNAs overlap with the 54 nucleotides of unknown origin. However, this sequence encompasses 28 nucleotides that are homologous to the most 5′ end of the 35S promoter (Figure 2B).

In short, these results indicate very stable and completely isogenic epialleles that differ only in their transcriptional activity. DNA methylation, suppressing chromatin marks, and sRNAs, are specifically enriched at the transcriptionally inactive epiallele; while the counterpart produces high transcript levels, lacks DNA methylation and sRNAs, and carries modifications characteristic of open chromatin (Figure 2C).

### Release of Silencing upon Sequence Rearrangement

**Figure 3:**
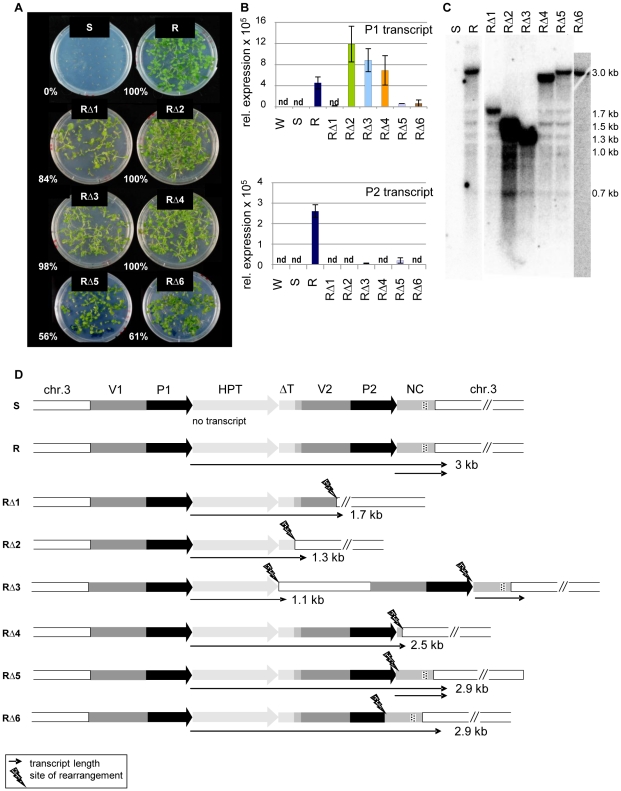
Sequence rearrangements after mutagenesis cause transcriptional activation of the silent epiallele. (A) Restored hygromycin resistance (percentage of resistant plants within all germinated seeds) of *cis*-mutants derived from line S after T-DNA mutagenesis, generation S4. (B) P1 and P2 transcript levels in generation S5 of *cis-*mutants determined by qRT-PCR. P1 was determined with primers within the HPT sequence, P2 with primers within the NC sequence. Due to the overlap, this might capture also some P1 templates. Normalization to S; nd: not detectable, reference gene *EIF4a* (At3g13920); error bars represent standard deviation of triplicate measurements. (C) Altered transcript length in *cis*-mutants (generation S4) compared to line R. Total RNA blot hybridized with an HPT probe. (D) DNA rearrangements determined after amplification and sequencing and transcript variation determined by 3′RACE and sequencing. Wild type (W); inactive (S) and active (R) epiallele, resistant *cis-*mutants derived from line S (RΔ1-6). The dashed part of the NC region indicates the overlap with small RNA reads homologous to P1/P2 (see Figure 2B).

In addition to the *trans-*acting mutants identified in a screen for restored *HPT* expression after mutagenesis of line S [22], we identified six hygromycin-resistant plants in which the mutant phenotype was genetically linked to the resistance gene itself (‘*cis*-mutations’, RΔ1-6). All these mutants produced progeny that could grow on hygromycin selection plates (Figure 3A), connected with restoration of variable amounts of P1 and P2 transcripts (Figure 3B). Northern blot analysis of *cis*-mutant RNA revealed P1 transcripts of smaller size in all *cis*-mutants compared to those from the active R line (Figure 3C). The length is reduced to different extents, indicating several independent mutational changes of the sequence. An extended northern blot analysis, with either total RNA or poly(A)-enriched RNA, showed that the P1 transcript in all lines besides RΔ6 is polyadenylated (Figure S6), likely due to a flanking sequence with some similarity to a polyA signal. While no P2 transcript from the second promoter is detectable in RΔ1, RΔ2, RΔ4, and RΔ6, there is a signal in RΔ3 and RΔ5, including in the poly(A) fraction (Figure S6C, S6D).

To characterize the P1 transcripts, and to identify the transcriptional termination sites in the *cis*-mutants, we performed 3′-RACE. We also analyzed the genomic DNA of all *cis*-mutants after amplification of the transgenic insert from genomic DNA and aligned DNA and RNA sequences (Figure 3D). This verified six different sequence rearrangements within the 3′ region: mainly deletions, but also one case of an inserted plant DNA fragment (RΔ3). The mutants RΔ1 and RΔ2 have both lost the duplicated promoter P2 and the NC sequence. The vector duplication was partially (RΔ1) or completely (RΔ2) deleted, as was part of the flanking plant sequence. The deletions in RΔ4, RΔ5, and RΔ6 did not or only partially affect the P2 promoter, and two of them maintain also the NC sequence. The rearrangement in RΔ3 is most complex: here, a 1243 bp plant DNA sequence derived from a position 1.2 kb upstream of the transgene location was inserted between the P1 transcript and the downstream vector fragment. In the mutants RΔ1, RΔ2, RΔ3, and RΔ4, the P1 transcripts are terminated at the (first) site of rearrangement, while the transcripts go beyond the breakpoints in RΔ5 and RΔ6. Only RΔ3 and RΔ5 are able to produce the P2 transcript, as in these cases, the P2 promoter is complete and the heterologous sequence downstream was only slightly affected by mutagenesis (Figure 3D). Nevertheless, the P2 transcript levels are much lower than in the R line (Figure 3B). Interestingly, there is no overlap between the deletions in all individual *cis*-mutants, but the rearrangements had either affected the second promoter copy (RΔ1, RΔ2, RΔ6), or the DNA template for the P2 transcript (RΔ1, RΔ2 and RΔ4), or the connection between both sequences (RΔ3, RΔ5).

All *cis*-mutants were tested for effects outside of the epiallele by analyzing the degree of genome-wide methylation at endogenous repeats and by introgressing a transcriptionally silent marker gene coding for β-glucuronidase from line L5, shown to be affected by other epigenetic mutations [27]–[28]. None of the *cis*-mutants changed the modification or expression of these markers (Figure S7). Therefore, it is unlikely that they have an effect outside of the epiallele.

**Figure 4:**
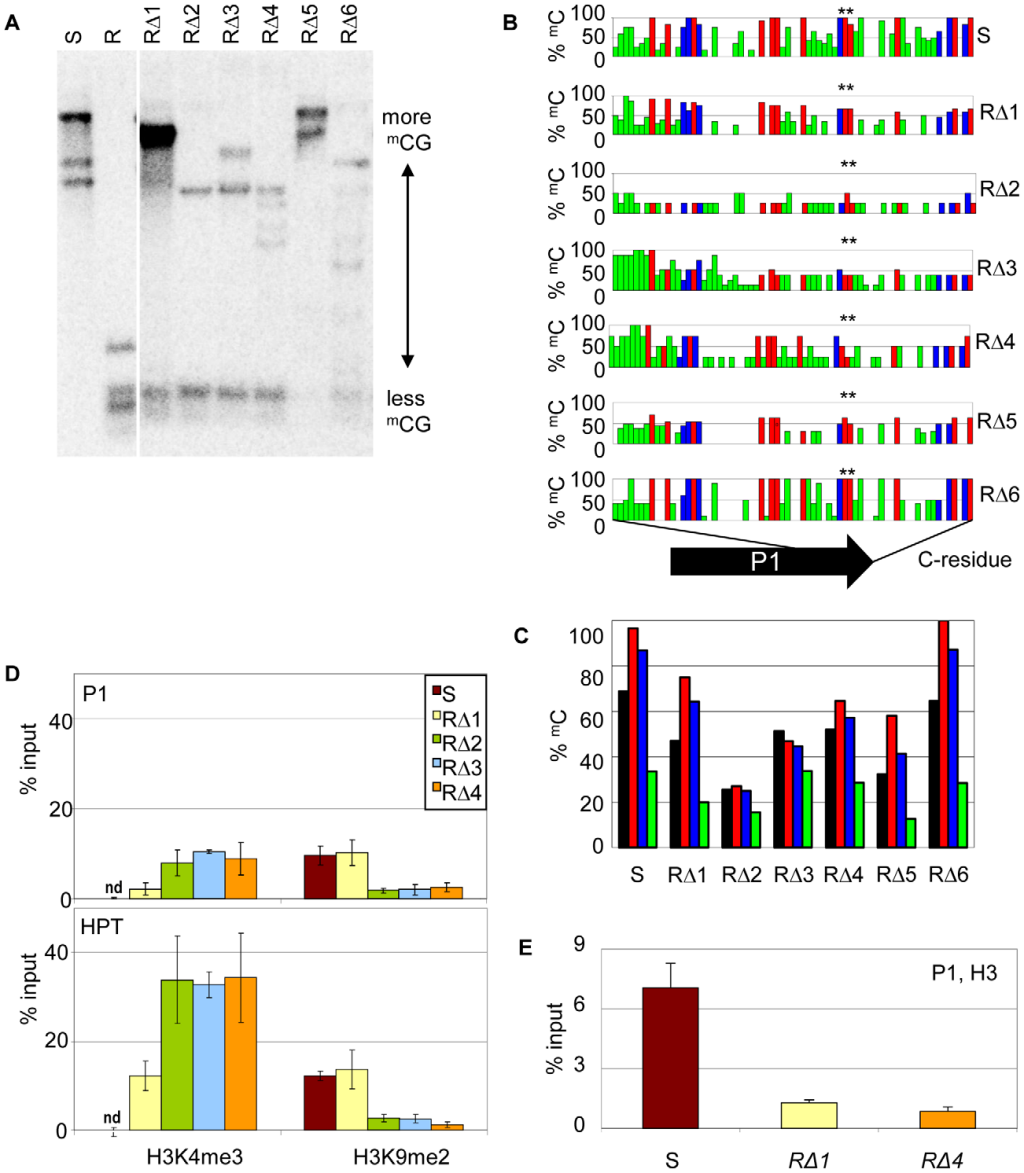
Sequence rearrangements change chromatin features. (A) Methylation analysis in three week-old seedlings of the *cis*-mutants. Genomic DNA was treated with *Hpa*II not cutting ^m^C^m^CGG, blotted and hybridized to a probe spanning the P1 promoter and the HPT gene. (B,C) DNA methylation in sequence-specific context (black: total ^m^C, red: ^m^CG, blue: ^m^CHG, green: ^m^CHH) at promoter P1 in the *cis-*mutants analyzed by bisulfite sequencing. Methylation at individual sites (B), summary of methylation across P1 (C). (D,E) Analysis of histone H3 at promoter P1 (amplicon 133 bp) and the HPT gene (amplicon 137 bp; primers see Table S4) in selected *cis-*mutants by chromatin immunoprecipitation. (D) Association with H3K4me3 and H3K9me2; nd: not detectable; (E) modification-independent precipitation. Inactive (S) and active (R) epiallele, resistant *cis-*mutants derived from line S (RΔ1-6).

Due to the hygromycin selection in the screen, all *cis*-mutants were expected to have a functional resistance marker gene. Indeed, the upstream promoter P1 and the HPT coding region were intact and identical in RΔ1-6 and hence potential new epialleles of the resistance gene. Therefore, we compared the chromatin state in this region. We found reduced DNA methylation levels in *cis*-mutants compared to S (Figure 4A), and a detailed bisulfite methylation analysis confirmed an overall reduction of DNA methylation in the promoter region of *cis*-mutants (Figure 4B, 4C). However, the degree of hypomethylation, and the distribution of the remaining methylated cytosine residues, do not support a direct and linear correlation with expression levels. Although RΔ2, RΔ3, and RΔ4 show the strongest reduction of CG methylation, especially at the transcription factor binding sites (Figure 4B, asterisk), and have expression levels comparable to R (Figure 3B), methylation in RΔ5 is similar to RΔ3 and RΔ4, although P1 transcript expression is much lower. Also, RΔ3 and RΔ4 have even gained CHH methylation in the 5′ region. Concomitant with the loss of DNA methylation, the modification specific for the silent state (H3K9me2) was changed in favor of the active mark (H3K4me3) in P1 and P1-transcribed regions, as demonstrated by ChIP (Figure 4D). One mutant (RΔ1) maintained a high level of H3K9me2 similar to that of the silent epiallele. Nonetheless, it also acquired a remarkable amount of H3K4me3, although less than other *cis*-mutants. Independent of the modifications, and similar to the resistant line, *cis*-mutants showed a decreased level of H3 association, indicating that the sequence rearrangements had also affected the nucleosome density (Figure 4E).

On the whole, the *cis*-mutants demonstrate that structural rearrangements can cause significant changes in transcriptional activation and chromatin configuration at the previously silent epiallele. These changes are surprisingly divergent and reflect specific effects of similar but not overlapping deletions.

### Stability of Silencing after Sequence Rearrangement

**Figure 5:**
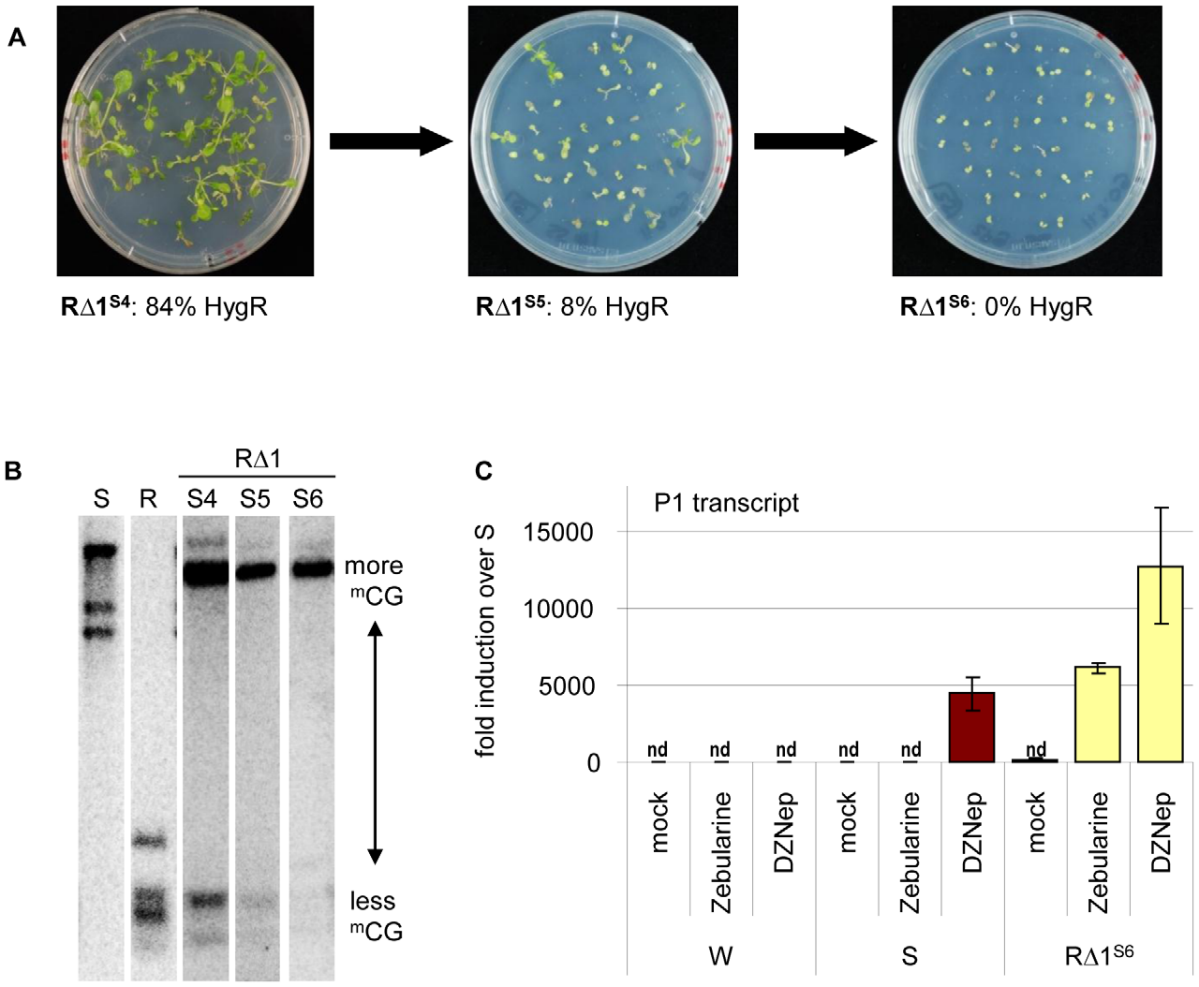
Different stability of reactivation after sequence rearrangement. (A) Hygromycin resistance in later generations of mutant RΔ1. (B) Progressive methylation in later generations of mutant RΔ1. Analysis as in Figure 4. (C) Effect of drug treatment (mock, 40 µM zebularine or 2 µM DZNep) on P1 transcript levels determined by qRT-PCR. Normalization to S; nd: not detectable, reference gene *EIF4a* (At3g13920); error bars represent standard deviation of triplicate measurements; wild type (W), line S (S) and *cis*-mutant RΔ1 in generation S6.

The extreme stability of R and S epialleles through many generations and in callus cultures raised the question of expression stability in the *cis*-mutants. Most structurally rearranged derivatives displayed similar stability and provided comparable hygromycin resistance over several generations of homozygous *cis*-mutants (S4 to S6 tested). RΔ2, RΔ3, and RΔ4 produced resistant progeny in consecutive generations. Resistance in RΔ5 and RΔ6 was lower in S4 (56% and 61%, respectively), but maintained this level up to S6. In contrast, RΔ1 plants that were clearly hygromycin-resistant in S4 (84%) generated partially sensitive S5 and fully sensitive S6 progeny (Figure 5A). This correlates well with the loss of unmethylated sites at the transgenic insert (Figure 5B), similar to gradual loss of resistance over 5 generations described for another marker gene [29]. The instability in RΔ1 does not correspond with additional sequence changes, as the same rearranged structure (Figure 3D) is maintained in subsequent generations. Rather, it correlates with the epigenetic state, since RΔ1 was characterized by the bivalent histone modifications (Figure 4D).

The re-silencing in generation S6 of RΔ1 allowed us to compare silencing maintenance at promoter 1 between this line and the S epiallele. We tested plants of both lines after growth in the presence of zebularine [reducing DNA methylation, 30] or DZNep [reducing histone methylation and also DNA methylation via SAHH]-[inhibition, 22,31]. Zebularine alone did not reactivate promoter P1 in line S, but in RΔ1^S6^, and DZNep-induced activation was twice as high in RΔ1^S6^ compared to S (Figure 5C). This indicates that S and RΔ1^S6^ differ in the stringency of silencing, either due to presence or absence of the P2 promoter and transcript, or to the lineage history of RΔ1^S6^ from a recently active state. The presence of the P2 promoter in RΔ3 - 6 and the expression of the P2 transcript in RΔ3 and 5, which do not cause re-silencing in later generations, make the latter explanation more likely.

## Discussion

The thorough analysis of the *HPT* transgene in its two opposite expression states has revealed sequence identity over the full length of the insertion, significant differences in chromatin modifications and few, but silencing-specific, small RNA molecules. Chromatin differences are restricted to the affected sequence, with no hint of genome-wide changes or modified localization of the genomic region within the nucleus. Together with heritability of the expression states over many generations, and their maintenance even upon de-differentiation, the data prove the transcriptionally active and the silenced version to be authentic epialleles. Their occurrence in *Arabidopsis*, the best studied model for epigenetic research in plants, and the easy assay for the selectable hygromycin resistance conferred by the active state, made this pair of epialleles convenient tools for studying maintenance and switching of epigenetic states.

After mutagenesis, we identified several hygromycin-resistant plants in which mutations in the epiallele sequence downstream of the *HPT* coding region had reactivated the previously silenced epiallele. Combining DNA and RNA sequence analysis and characterization of chromatin modifications, we found that these structural changes of the DNA sequence caused substantial upstream changes in chromatin and transcriptional activity. Beyond the complex and mutually dependent interplay of chemical modifications of the DNA and the associated histones, and longer and small, coding and non-coding RNAs described in numerous cases, the results presented here have shown that even small and non-overlapping modifications of the genomic template, outside of the promoter and open reading frame, can modify transcription and chromatin states in the vicinity. These changes are not minor: the bacterial gene *HPT* coding for hygromycin phosphotransferase is a selectable marker gene applied in numerous plant transformation experiments [32], but plants need a significant amount of *HPT* transcript to produce enough protein to detoxify the antibiotic. Minor reactivation in the background of some epigenetic mutants tested in a reverse genetic approach (data not shown) was not sufficient. Therefore, the stringent assay for restored hygromycin resistance required a substantial change, as in the case of the trans-acting mutants from the same screen that revealed a double lock of two simultaneous chromatin modifications [22]. *HPT* expression levels are indeed similar between *cis*-and *trans*-acting mutants.

Although the transgenic marker allowed this convenient selection for drastic epigenetic switches, without affecting plants under non-selective conditions, it could have been considered not representative for other, plant-endogenous or general cases. However, a recent publication [33] describes an interesting mutation that affects expression of the gene for nodulation factor SUNN in *Medicago truncatula*. The mutation is closely linked to the *SUNN* gene, acts only in *cis* but does not change the DNA sequence of the *SUNN* gene itself. Although the nature of this mutation is not yet identified, it could exert its effect in a similar way to the *cis*-mutants described here, especially since the ‘like sunn supernodulator’ mutant phenotype is occasionally unstable, like the hygromycin resistance in RΔ1, 5, and 6. Other examples may be found upon further inspection of natural transcript level variation between regions with very similar gene sequences in plants [e.g. 8] or in the connection between chromatin structure and trinucleotide repeat expansion in mammals [for review 34].

Transcriptional gene silencing is often associated with the presence of homologous sequences in the genome [e.g. 35]–[37], and intentional rearrangements from complex inserts to single copies by site-specific recombinase eliminate silencing [e.g. 38]. Therefore, when we started the analysis of the sequence changes in the *cis*-mutants, we were expecting a clear dependence of reactivation on loss of the duplicated region. This is not the case, since all *cis*-mutants, with the exception of RΔ2, still retain some duplicated regions. Also against expectation, a loss of the non-coding sequence homologous to the most abundant small RNAs is not a prerequisite for reactivation (RΔ3, RΔ5, and RΔ6). Furthermore, a loss of the small transcript starting from the P2 promoter is not necessary (RΔ3 and RΔ5), although its level in these mutants is not as high as in R plants. It should be kept in mind that neither the tandem sequence duplications, nor either of the two transcripts, are sufficient to initiate silencing, since R plants (with the complete insert and substantial transcription from P1 and P2) are fully resistant and stable. This is distinct from the *FWA* gene where tandem repeats are necessary and sufficient for silencing and DNA methylation [39]. Considering the lack of DNA methylation and small RNAs at the *HPT* insert in R plants, it is possible that the initial steps of silencing do not occur, are not efficient enough to start the reinforcing mechanism [39], or are inhibited by efficient transcription [40]. However, such conditions must have been overruled on the rare occasions that produced the silent epiallele in the first place.

The deletions in the different *cis*-mutants do not overlap in a specific region, and the smallest change is the loss of just 65 bp (RΔ5). Apparently, rather than affecting a specific sequence, the rearrangements change the overall organization at this locus. These changes can have variable consequences for the upstream promoter, causing either decisive, stable epigenetic switches (RΔ2, RΔ3, RΔ4) or leading to ambivalent states that can later fall back into silencing (RΔ1). How such small genetic heterogeneity, that does not affect coding or regulatory regions, can cause extreme epigenetic diversity at a promoter elsewhere remains an open question. The sequence changes could exert their effect by modifying the distance to flanking regulatory regions, the nucleosome arrangement or density, the association with DNA-binding molecules, or any higher order structure within the DNA. It is clearly different from the ‘spreading’ effect of silencing often associated with RdDM [41]–[42]: it causes activation (not silencing), goes against (not along with) the direction of transcription, and the most abundant of the relatively few small RNAs does not match the affected sequence of the upstream promoter. The results emphasize the mutual dependence between genetic and epigenetic factors, while indicating that these do not necessarily act at overlapping genomic sites. Similar effects might explain some of the associated changes in gene expression in the vicinity of small or large sequence modifications by transposon or recombination events. One example at a similar distance might be the transposon-dependent loss and gain of DNA methylation and inverse gene expression regulating sex determination in melon, at a site just 1.5 kb away from the insertion/excision site [43].

The relatively high number of *cis*-mutants in the screen was plausible in retrospective: mutations outside of the epiallele released silencing only if they reduce two epigenetic marks simultaneously. This is achieved by a few special mutations [22] or theoretically by rare double mutations and explains the low number of *trans*-acting mutants. In the study here, the genetic changes were found after mutagenesis by *Agrobacterium*-mediated T-DNA transformation [22], although none of the *cis*-mutations was connected with an integrated fragment of the incoming T-DNA. T-DNA transformation is also known to create mutations unlinked, or independent, from the site of integration [44] and can cause complex chromosome rearrangements [45]–[46]. Successful, and possibly also attempted, integrations occur at sites of microhomologies between T-DNA and plant DNA [47]–[48]. The incoming T-DNA [49] has some homology with the terminator sequences in the epiallele (ΔT), and in fact, the deletion sites in two *cis*-mutants (RΔ2, RΔ3) are near, or in, this sequence. The other deletions are close to promoter copy P2 that has no homology with the T-DNA, but potentially reflect a recombination hotspot in the 35S promoter sequence [50]. Alternatively, the double strand breaks connected with completed or aborted integration might stimulate repair via homologous recombination between the duplicated sequences of the epiallele (RΔ3). This would indeed have selected for 3′ rearrangements since those affecting the upstream copy are likely to lose the functional *HPT* cassette.

**Figure 6:**
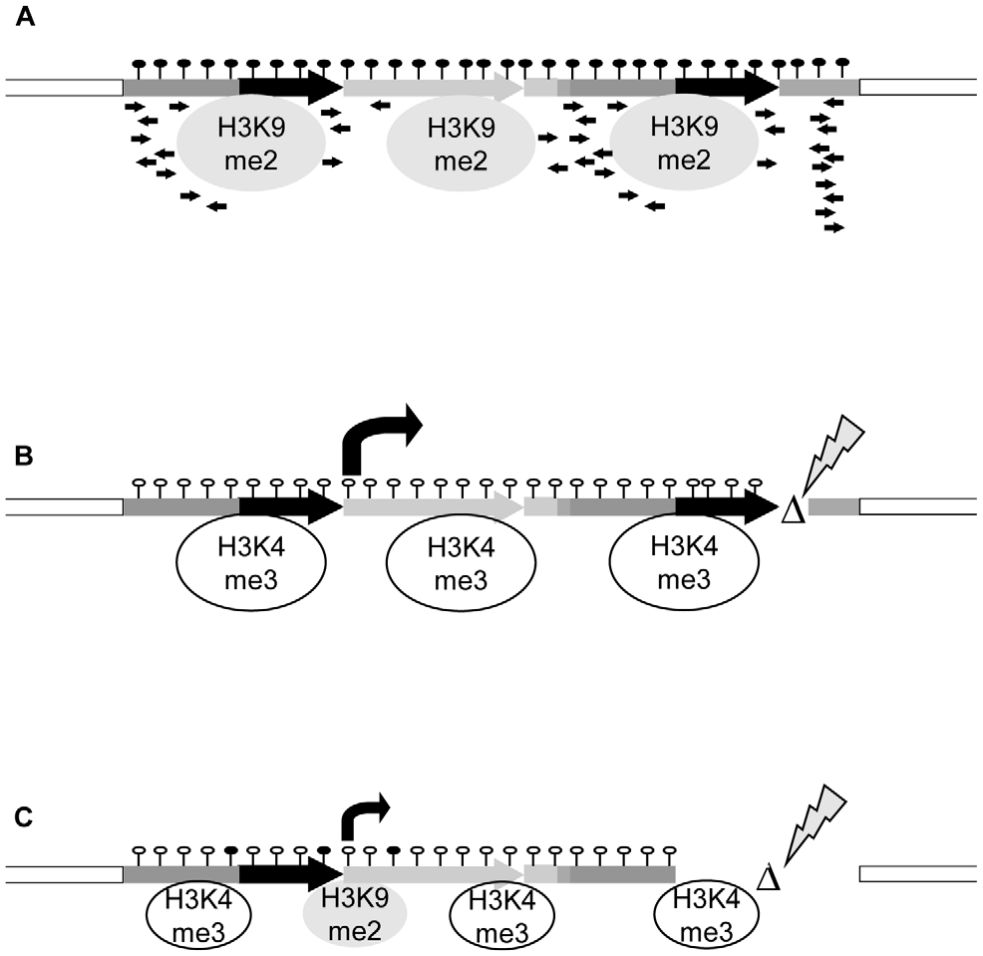
Model for transcriptional regulation of epialleles. (A) The silent state is maintained by interplay of DNA hypermethylation, repressive histone modifications, lack of activating marks, presence of sRNAs and structure of the controlled gene, all mutually reinforcing the block of transcription. (B,C) Release of silencing can occur after structural rearrangements. (B) Even minor changes, like in *cis-*mutant RΔ4, can trigger a switch to high transcript levels, drastic changes of chromatin features and stable genetic transmission. (C) A major rearrangement like in *cis-*mutant RΔ1, although deleting the sRNA-producing region, does not necessarily result in stable switches. Transcript levels are lower; exchange of histone marks is incomplete and not stable in subsequent generations. This points to a significant role of sequence or gene structure, possibly by different secondary structure or nucleosome positioning, in stability of epialleles.

All together, the R and S epialleles described here provide an example of identical DNA sequences with converse expression states and specific epigenetic configuration that are faithfully transmitted to progeny. However, sequence changes in the vicinity of the silent epiallele can induce an epigenetic switch to the opposite state. These can have different degrees of stability, depending on the complex interplay between the nature of the sequence alteration, the consequences for transcription and transcripts, and the chromatin organization (Figure 6). This also illustrates a tight dependence of epigenetic regulation on local structures and makes it likely that DNA rearrangements can potentially change or induce new epialleles outside the affected region.

## Materials and Methods

### Plant Material, Growth, and Chemical Treatments

*Arabidopsis thaliana* lines with R and S epialleles in accession Zürich and mutagenesis of line S were described previously [20], [22]. Stratified seeds were surface-sterilized with 5% sodium hypochlorite and 0.05% Tween-80 for 6 min, washed and air-dried overnight. Sterilized seeds were germinated and grown in Petri dishes containing agar-solidified germination medium (GM) in growth chambers under 16 h light/8 h dark cycles at 21°C. For drug treatments, seeds were sown and plants grown on GM plates with hygromycin (10 µg/ml, Calbiochem), zebularine (40 µM, Sigma) or 3-deazaneplanocin (DZNep, 2 µM, donated by Dr. Victor Marquez) under the conditions described above.

### Nucleic Acid Isolation and Gel Blot Analysis

Genomic DNA was isolated from 3 week-old seedlings using either DNeasy Plant Mini Kit (Qiagen) or Phytopure (Amersham), following the manufacturers' protocols, except that genomic DNA was eluted in sterile water. Total RNA extraction from 3 week-old seedlings was performed with RNeasy Plant Mini Kit (Qiagen) including an on-column DNase I digest (Qiagen). For Southern blot analysis, 10 µg of genomic DNA were digested overnight with 20 U restriction enzymes. For methylation-specific Southern blot analysis, the methylation-sensitive restriction enzymes (*Hpa*II, blocked by ^m^CG and ^m^CHG, and *Msp*I, blocked only by ^m^CHG) were used. Digested samples were electrophoretically separated on 1.2% TAE agarose gels, depurinated for 10 min in 250 mM HCl, denaturated for 30 min in denaturation solution containing 0.5 M NaOH and 1.5 M NaCl and neutralized twice in 0.5 M Tris, 1.5 M NaCl and 1 mM EDTA at pH7.2 for 15 min. For northern blot analysis of total and poly(A) RNA, 5 µg of RNA were denatured with 15% glyoxal and 50% DMSO for 1 h at 50°C and separated using 1.5% agarose gels in 10 mM sodium phosphate buffer pH7 in a Sea2000 circular flow electrophoresis chamber (Elchrom Scientific). DNA and RNA gels were blotted onto Hybond N+ (Amersham) membranes overnight with 20× SSC, washed and UV-crosslinked using a Stratalinker (Stratagene). Hybridization was performed as described [51]. Radioactively labeled sequence-specific probes were synthesized from 25 ng of DNA using the Rediprime labeling kit (Amersham) and 50 µCi dCTP-α-^32^P (Amersham or Hartmann Analytic) and purified on G50 Probequant (Amersham) columns. Signals were detected with phosphoimager screens (Bio-Rad) and scanned with a Molecular Imager FX (Bio-Rad).

### Rapid Amplification of cDNA 3′ Ends

3′-RACE was performed with the SMART RACE cDNA Amplification Kit (Clontech) according to the instructions. Total RNA (700 ng) was treated with DNaseI (Fermentas), then reverse-transcribed with RevertAidRT (Fermentas) with 3-RACE A primer (5–AAGCAGTGGTATCAACGCAGAGTAC(T)30V N–3) in a 20 µl reaction. Two µl of cDNA reaction were used as template in 3′-RACE PCR. For this, Advantage 2 PCR Kit (Clontech) was used according to instructions. A control primer (Actin, Act2F primer: 5-GCCATCCAAGCTGTTCTCTC-3) and gene-specific primers were used in combination with UniA_45 (5–CTAATACGACTCACTATAGGGCAAGCAGTGGTATCAACGCAGAGT–3).

### Reverse Transcription PCR and Quantitative Real-Time PCR

RNA samples were treated with DNase I (MBI Fermentas) for 30 min at 37°C to remove residual DNA contamination. The reaction was inactivated by addition of EDTA and incubation at 65°C for 10 min. Reverse transcription was performed on 1 µg of RNA with 0.2 µg of random hexamer primers (MBI Fermentas) using 1 U RevertAid H Minus M-MuLV-RTase (MBI Fermentas) in the presence of 20 U RiboLock Ribonuclease inhibitor at 42°C for 1.5 h. Real time PCR analysis was performed with the 2× SensiMix Plus SYBR & Fluorescein Kit (Quantace) protocol using an iQ5 Real-Time-PCR System (BioRad Laboratories). The obtained Ct values were analyzed with the iQ5 Optical System Software Version 2.0 (Bio-Rad), applying the mathematical model for relative quantification in Excel (Microsoft) as described [52]. All primer sequences are listed in Table S4.

### Bisulphite Conversion, Sequencing, and Evaluation

After treatment with RNase A and proteinase K, 1–2 µg of genomic DNA were digested overnight with *Bam*HI (MBI Fermentas). Subsequent bisulphite conversion was carried out using the Epitect Conversion Kit (Qiagen) and controlled for completion as described [21], [53]. Converted DNA was used for PCR amplification. PCR-amplified DNA was cloned using pGEM-Teasy (Promega) and ligation mixes transformed into DH5α cells (Invitrogen) and sequenced by terminal-labeling using BigDye Terminator v3.1 (Applied Biosystems). The sequence information obtained was analyzed with CyMATE, www.gmi.oeaw.ac.at/cymate [54], and Excel (Microsoft).

### Chromatin Immunoprecipitation

ChIP was performed as described (http://mescaline.igh.cnrs.fr/EpiGeneSys/www/images/protopdf/p13.pdf) using 3 week-old seedlings. The chromatin was immuno-precipitated with antibodies to histone H3 (Abcam, ab1791), H3K4me3 (Upstate, 07-473), H3K9me2 (T. Jenuwein 4677 and Abcam ab1220), and H3K27me2 (Upstate, 07-473). Immunoprecipitated DNA was purified using a Qiagen PCR Purification Kit and eluted in 50 µl of EB buffer. Quantitative real-time PCR was carried out in a total reaction volume of 25 µl and qPCR conditions were according to the 2× SensiMix Plus SYBR & Fluorescein Kit (Quantace) protocol using an iQ5 Real-Time-PCR System (BioRad Laboratories). qPCR data were evaluated as a ratio to input DNA [55].

### sRNA Isolation, Library Generation, and Bioinformatic Analysis

Small RNA was isolated from either pooled inflorescences or seedlings (21 days old) using the mirVana miRNA Isolation Kit (Ambion). Small RNA libraries were generated as previously described [56] and sequenced using the Illumina G2 platform. After clipping the adapter sequence by vectorstrip software from EMBOSS package [57], small RNA reads were screened for homology with the epiallele sequence using bowtie [58], allowing only perfect matches (Table S3). Reads homologous to tRNA, rRNA, snRNA, snoRNA, mitochondrial RNAs, and chloroplast RNAs were removed by custom Perl scripts. The total number of reads that mapped to a certain region was computed as sum of 1/N_i (N_i is the number of times the read i was mapped). It was then normalized to indicate the number of each read per million bp (adapted from the RPKM concept in RNA-Seq, [59]. A threshold of 10 reads was chosen for any sequence to be taken into account. For the epiallele region, the normalized number of mapped reads was computed at single bp scale. For a more detailed view on a selected region, the analysis was performed with SiLoMa [60].

Additional methods are described in Text S1.

## Supporting Information

Figure S1 Schematic representation of the origin of the epialleles. Protoplast culture of transgenic, diploid and hygromycin-resistant line C [25] and regeneration resulted in tetraploid plants without (red) or with (blue) hygromycin resistance. The tetraploids were diploidized by repeated backcrossing to diploid wild type and subsequent selfing to generate homozygotes.
(TIF)

Figure S2 Histone modifications within epialleles and flanking regions. Histone H3 modifications were analysed at eight positions by chromatin immunoprecipitation using antibodies against H3K4me3, H3K9me2 and H3K27me2. S, inactive epiallele; R, active epiallele; W, wild type.
(TIF)

Figure S3 Localization of epialleles in interphase nuclei. To investigate whether the epigenetic state had any influence on the location of the epialleles within the nucleus we performed fluorescence *in situ* hybridization (FISH) on flow-sorted interphase nuclei from S and R lines and wild type. We used different BAC probes on chromosomes 1 and 3 (A). The genomic location of the epiallele and two control regions with equal distance to the telomeres were marked by two differently labelled neighboring BAC clones each. We determined the percentage of nuclei with one (B) or two (C) signals, indicating pairing or non-pairing of corresponding regions (D). We further examined the intranuclear localization (E) and the co-localization with centromeric heterochromatin (180 bp repeats, E) of the epialleles. No significant differences between S, R, and wild type were observed, indicating that the expression state did not modify the position within the nucleus or the association with heterochromatin. Bar = 5 µm. S, inactive epiallele; R, active epiallele; W, wild type.
(TIF)

Figure S4 Maintenance of epigenetic modifications at epialleles in callus culture. (A) DNA methylation analysis at promoters P1 and P2 in callus tissue by bisulfite sequencing representing total (^m^C) and sequence context-specific (^m^CG, ^m^CHG, ^m^CHH) methylation in plant tissue and dedifferentiated callus. (B) Methylation analysis of callus tissue DNA treated with *Hpa*II not cutting ^m^C^m^CGG, blotted and hybridized to a probe spanning the P1 transcript. (C) Histone H3 modifications at promoter duplications analysed in callus tissue by chromatin immunoprecipitation using antibodies against H3K4me3, H3K9me2 and H3K27me2. S, inactive epiallele; R, active epiallele; W, wild type.
(TIF)

Figure S5 Analysis of antisense transcripts. The overlap between the short and the long transcript from the two promoters suggested a search for non-coding RNAs involved in silencing maintenance. To investigate whether the non-coding sequence (NC) downstream of P2 could have served as a promoter to produce antisense RNA from the epiallele, we cloned the NC fragment in both orientations in front of a *GUS* reporter gene, replacing the P35S promoter in vector pCBK04. We then tested the constructs by transient transformation via *Agrobacterium tumefaciens* of an *Arabidopsis* Col-0 cell suspension culture and screened for GUS expression (A). None of the constructs gave any indication of GUS expression, making a promoter-like function of the NC sequence unlikely. Further, we analyzed potential antisense transcripts by northern blot hybridization with labeled strand-specific oligonucleotides homologous to different regions (P1/P2, HPT, V1/V2, NC) of the epiallele (B). Control sense and anti-sense RNA included in the blots were generated by in vitro T7 or SP6 polymerase transcription of the respective sequences cloned in the pGEM-T easy vector (Promega). No specific antisense RNA from the epiallele could be detected. This negative result was further confirmed for S and R lines by RT-PCR with primers at three different positions (data not shown). S, inactive epiallele; R, active epiallele; W, wild type.
(TIF)

Figure S6 Analysis of polyadenylation. Analysis of polyadenylation by northern blot hybridization of total (A,C,E) and poly(A)-enriched (B,D,F) RNA from *cis*-mutants in comparison to S (inactive epiallele) and R (active epiallele). * RNA sample degraded. (A,B) Probe specific for P1 transcript (HPT, Figure 1A). (C,D) Probe recognizing also P2 transcript (NC, Figure 1A). (E,F) U6 probe as a control for poly(A)-enrichment, excluding contamination with total RNA.
(TIF)

Figure S7 Analysis of *cis*-mutants for effects on global methylation and trans-activation. (A) Global cytosine methylation levels were measured by HPLC after hydrolysis of genomic DNA. (B) Line 5 with a transcriptionally silent GUS gene was crossed with the c*is*-mutants and F2 plant homozygous for the mutations analyzed for GUS expression. S, inactive epiallele; R, active epiallele; W, wild type; *ddm1*, mutant known to reduce global methylation and to trans-activate GUS.
(TIF)

Table S1 Normalization of small RNA libraries using Bowtie.
(PPT)

Table S2 Distribution of small RNAs.
(PPT)

Table S3 Summary of small RNAs reads in epialleles.
(PPT)

Table S4 Primer list.
(PPT)

Text S1 Supplemental methods and references.
(DOC)
