# Supplementary figures and images for: Genetic Rearrangements Can Modify Chromatin Features at Epialleles
Source: PLoS Genet. 2011 Oct 20;7(10):e1002331. doi: 10.1371/journal.pgen.1002331 (PMC3197671; doi:10.1371/journal.pgen.1002331)

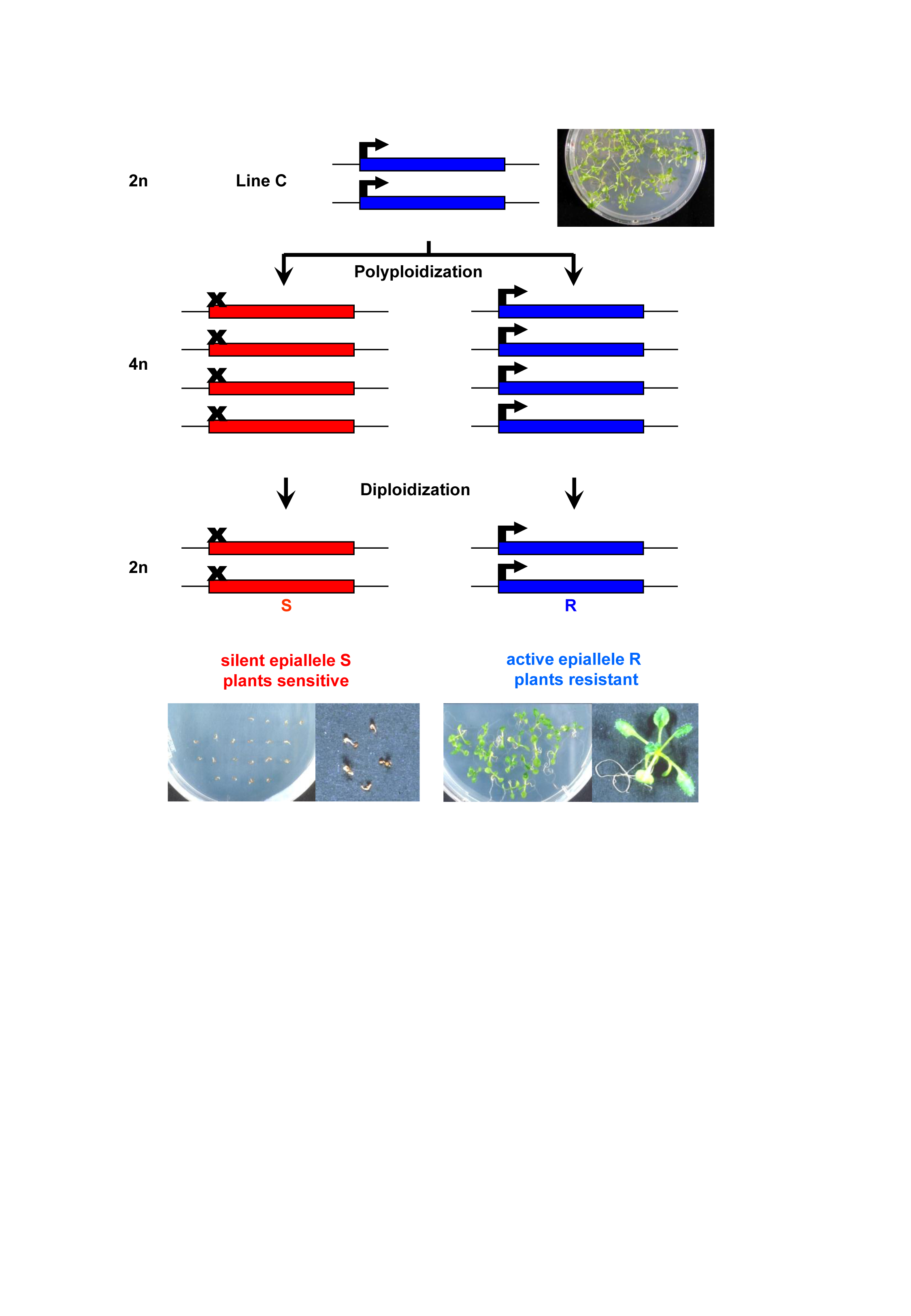

Supplement: Figure S1 — Schematic representation of the origin of the epialleles. Protoplast culture of transgenic, diploid and hygromycin-resistant line C [25] and regeneration resulted in tetraploid plants without (red) or with (blue) hygromycin resistance. The tetraploids were diploidized by repeated backcrossing to diploid wild type and subsequent selfing to generate homozygotes. (TIF) [file pgen.1002331.s001.tif]

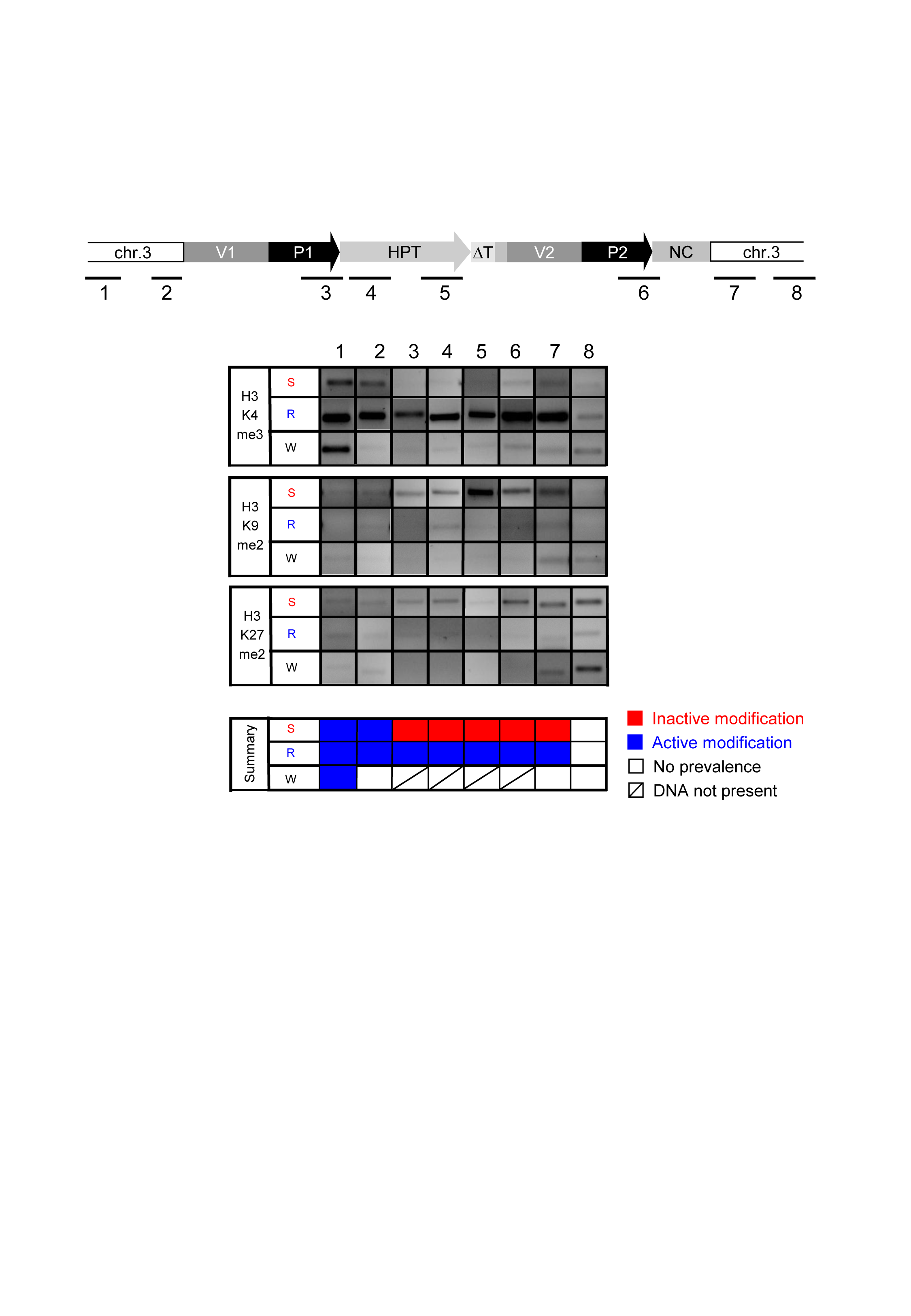

Supplement: Figure S2 — Histone modifications within epialleles and flanking regions. Histone H3 modifications were analysed at eight positions by chromatin immunoprecipitation using antibodies against H3K4me3, H3K9me2 and H3K27me2. S, inactive epiallele; R, active epiallele; W, wild type. (TIF) [file pgen.1002331.s002.tif]

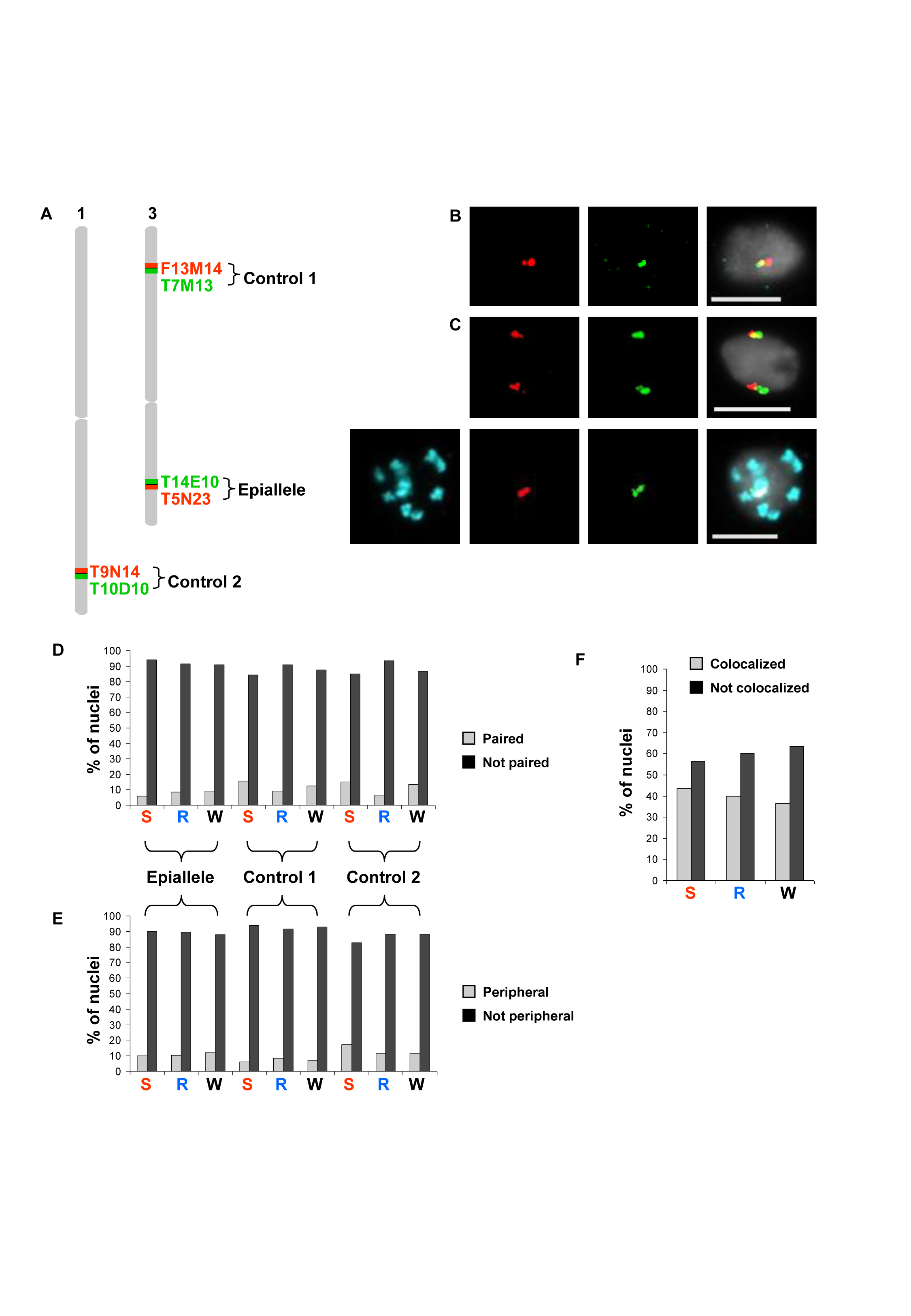

Supplement: Figure S3 — Localization of epialleles in interphase nuclei. To investigate whether the epigenetic state had any influence on the location of the epialleles within the nucleus we performed fluorescence in situ hybridization (FISH) on flow-sorted interphase nuclei from S and R lines and wild type. We used different BAC probes on chromosomes 1 and 3 (A). The genomic location of the epiallele and two control regions with equal distance to the telomeres were marked by two differently labelled neighboring BAC clones each. We determined the percentage of nuclei with one (B) or two (C) signals, indicating pairing or non-pairing of corresponding regions (D). We further examined the intranuclear localization (E) and the co-localization with centromeric heterochromatin (180 bp repeats, E) of the epialleles. No significant differences between S, R, and wild type were observed, indicating that the expression state did not modify the position within the nucleus or the association with heterochromatin. Bar = 5 µm. S, inactive epiallele; R, active epiallele; W, wild type. (TIF) [file pgen.1002331.s003.tif]

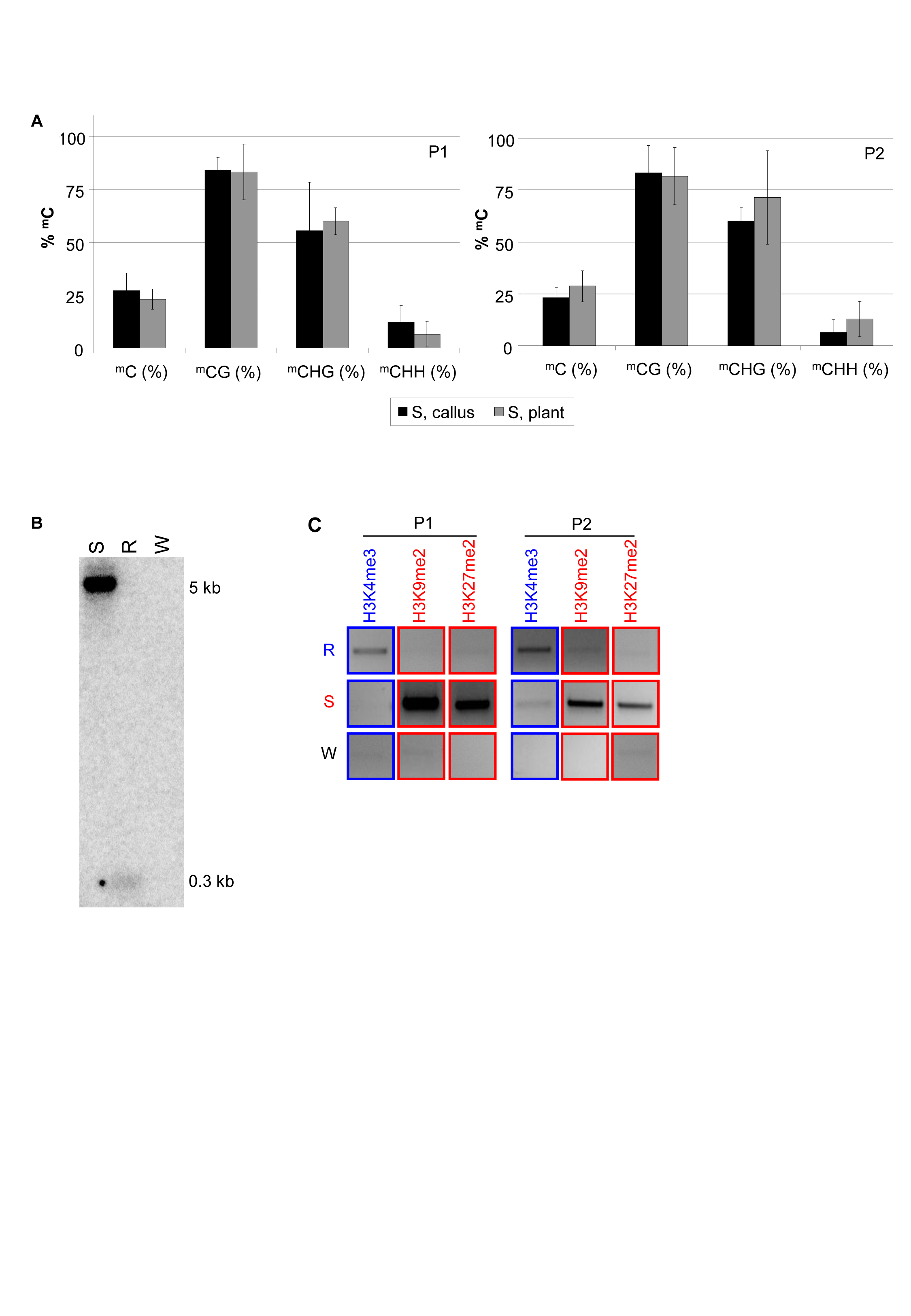

Supplement: Figure S4 — Maintenance of epigenetic modifications at epialleles in callus culture. (A) DNA methylation analysis at promoters P1 and P2 in callus tissue by bisulfite sequencing representing total (mC) and sequence context-specific (mCG, mCHG, mCHH) methylation in plant tissue and dedifferentiated callus. (B) Methylation analysis of callus tissue DNA treated with HpaII not cutting mCmCGG, blotted and hybridized to a probe spanning the P1 transcript. (C) Histone H3 modifications at promoter duplications analysed in callus tissue by chromatin immunoprecipitation using antibodies against H3K4me3, H3K9me2 and H3K27me2. S, inactive epiallele; R, active epiallele; W, wild type. (TIF) [file pgen.1002331.s004.tif]

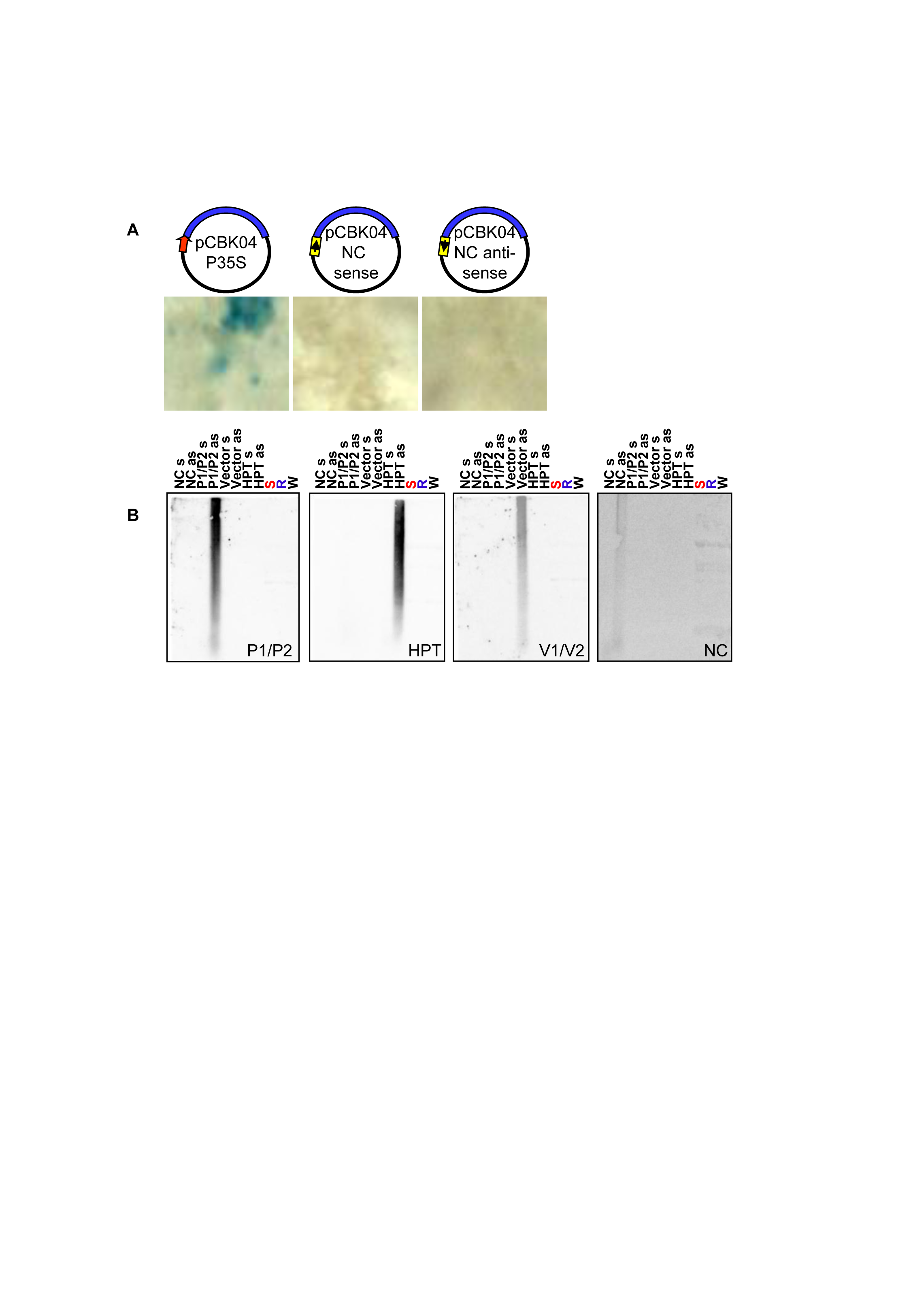

Supplement: Figure S5 — Analysis of antisense transcripts. The overlap between the short and the long transcript from the two promoters suggested a search for non-coding RNAs involved in silencing maintenance. To investigate whether the non-coding sequence (NC) downstream of P2 could have served as a promoter to produce antisense RNA from the epiallele, we cloned the NC fragment in both orientations in front of a GUS reporter gene, replacing the P35S promoter in vector pCBK04. We then tested the constructs by transient transformation via Agrobacterium tumefaciens of an Arabidopsis Col-0 cell suspension culture and screened for GUS expression (A). None of the constructs gave any indication of GUS expression, making a promoter-like function of the NC sequence unlikely. Further, we analyzed potential antisense transcripts by northern blot hybridization with labeled strand-specific oligonucleotides homologous to different regions (P1/P2, HPT, V1/V2, NC) of the epiallele (B). Control sense and anti-sense RNA included in the blots were generated by in vitro T7 or SP6 polymerase transcription of the respective sequences cloned in the pGEM-T easy vector (Promega). No specific antisense RNA from the epiallele could be detected. This negative result was further confirmed for S and R lines by RT-PCR with primers at three different positions (data not shown). S, inactive epiallele; R, active epiallele; W, wild type. (TIF) [file pgen.1002331.s005.tif]

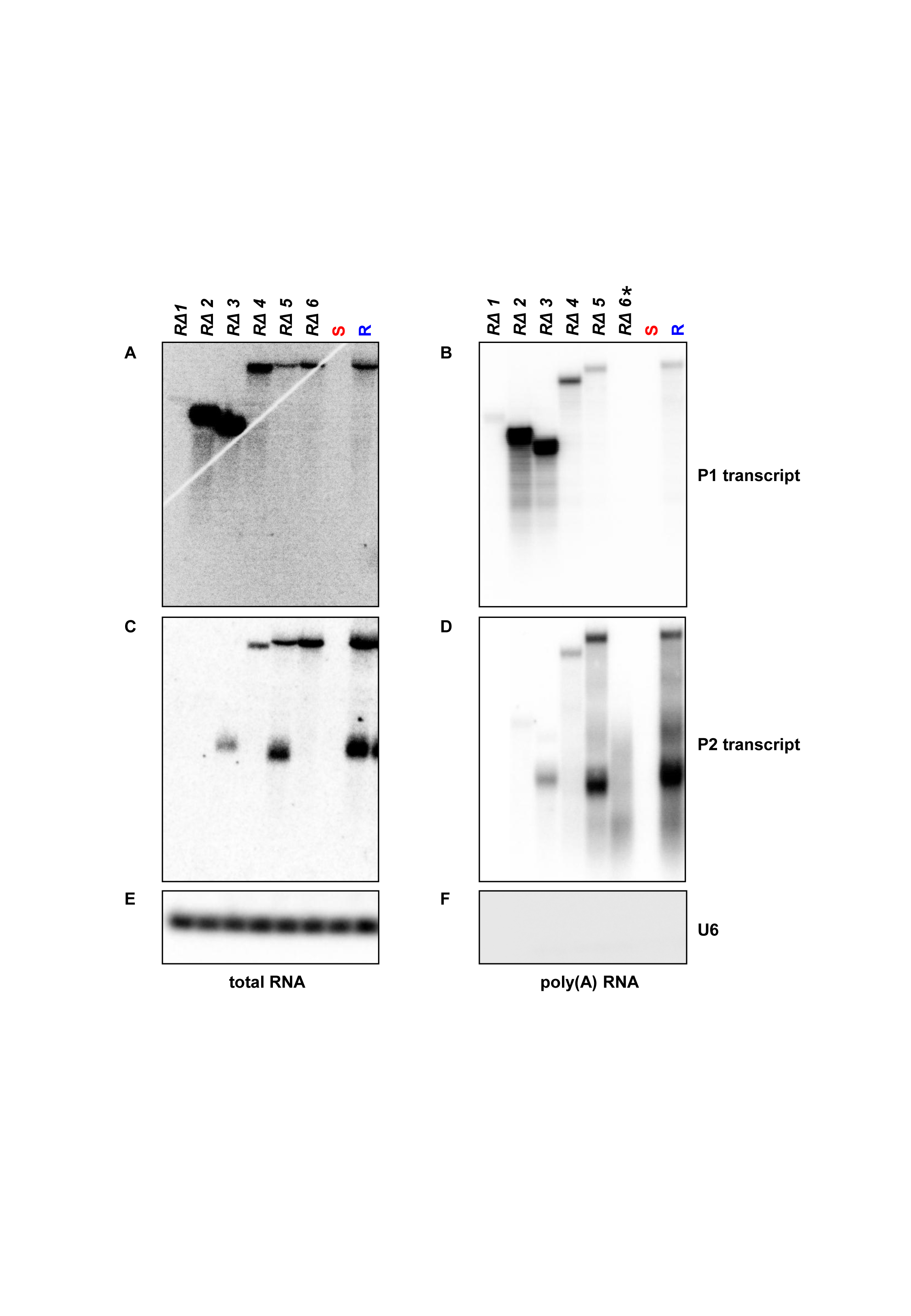

Supplement: Figure S6 — Analysis of polyadenylation. Analysis of polyadenylation by northern blot hybridization of total (A,C,E) and poly(A)-enriched (B,D,F) RNA from cis-mutants in comparison to S (inactive epiallele) and R (active epiallele). * RNA sample degraded. (A,B) Probe specific for P1 transcript (HPT, Figure 1A). (C,D) Probe recognizing also P2 transcript (NC, Figure 1A). (E,F) U6 probe as a control for poly(A)-enrichment, excluding contamination with total RNA. (TIF) [file pgen.1002331.s006.tif]

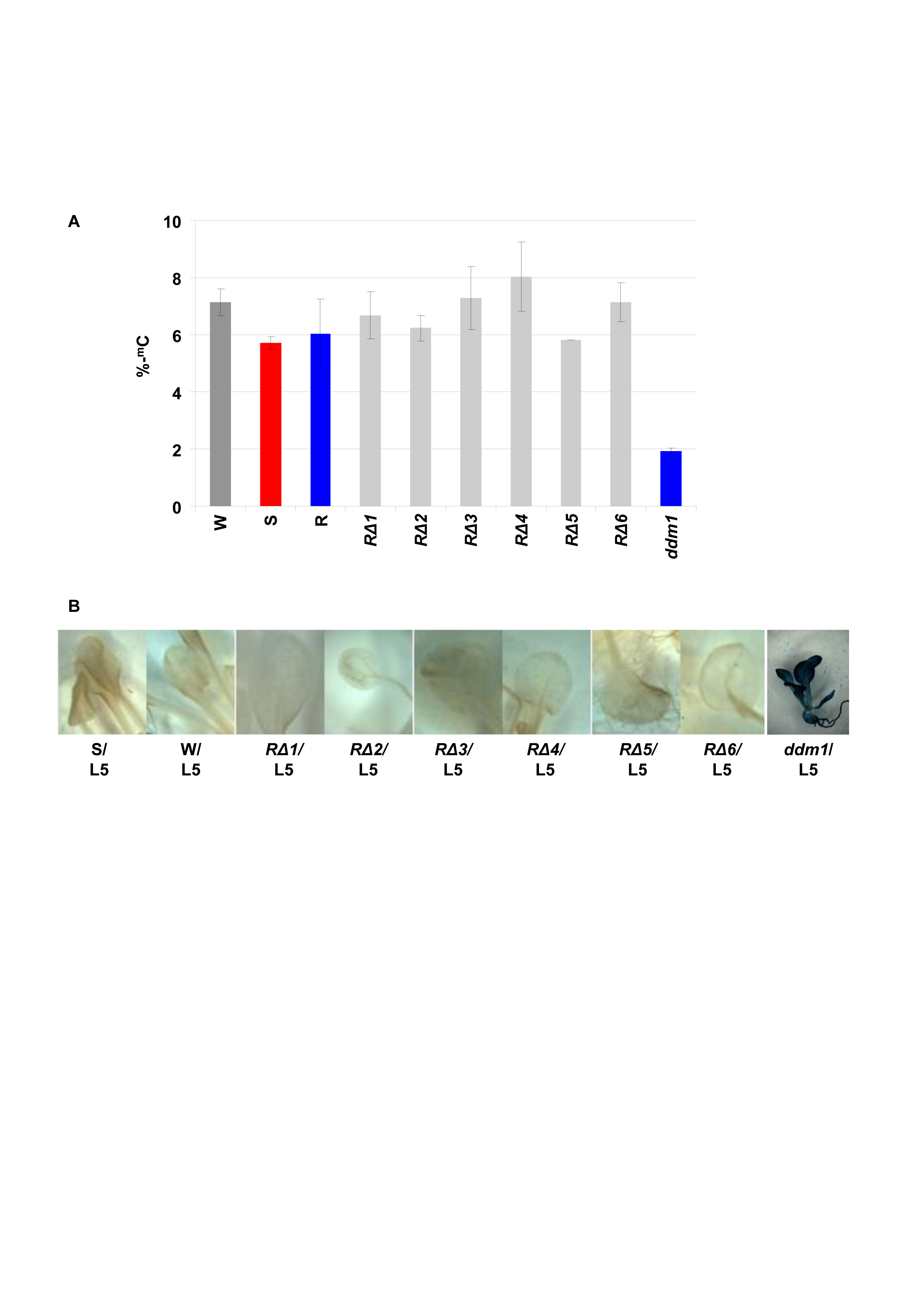

Supplement: Figure S7 — Analysis of cis-mutants for effects on global methylation and trans-activation. (A) Global cytosine methylation levels were measured by HPLC after hydrolysis of genomic DNA. (B) Line 5 with a transcriptionally silent GUS gene was crossed with the cis-mutants and F2 plant homozygous for the mutations analyzed for GUS expression. S, inactive epiallele; R, active epiallele; W, wild type; ddm1, mutant known to reduce global methylation and to trans-activate GUS. (TIF) [file pgen.1002331.s007.tif]

## Slide 1
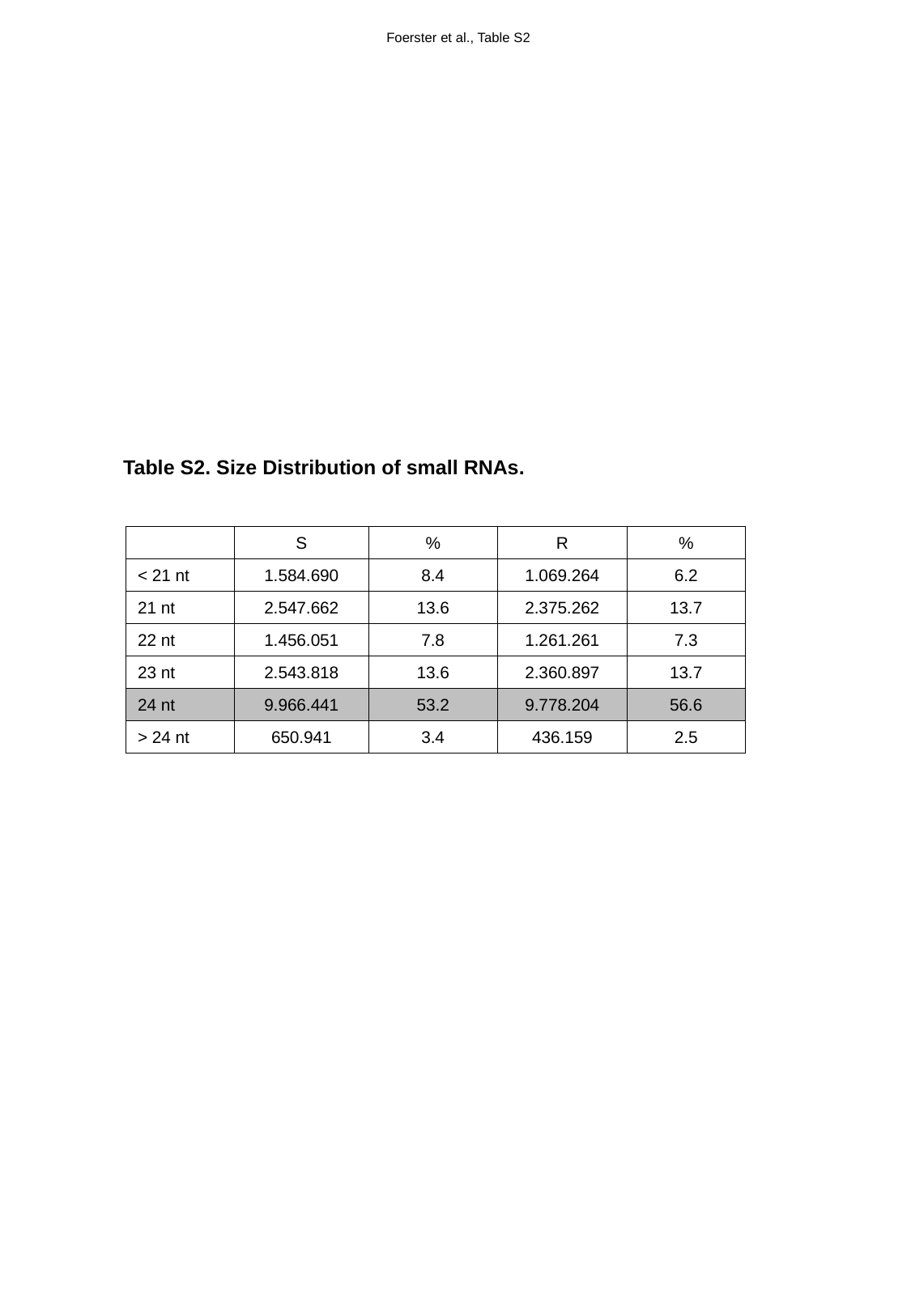

Foerster et al., Table S2
Table S2. Size Distribution of small RNAs.
| | S | % | R | % |
| --- | --- | --- | --- | --- |
| < 21 nt | 1.584.690 | 8.4 | 1.069.264 | 6.2 |
| 21 nt | 2.547.662 | 13.6 | 2.375.262 | 13.7 |
| 22 nt | 1.456.051 | 7.8 | 1.261.261 | 7.3 |
| 23 nt | 2.543.818 | 13.6 | 2.360.897 | 13.7 |
| 24 nt | 9.966.441 | 53.2 | 9.778.204 | 56.6 |
| > 24 nt | 650.941 | 3.4 | 436.159 | 2.5 |

Supplement: Table S2 — Distribution of small RNAs. (PPT) [file pgen.1002331.s009.ppt]
