# Supplementary material for: Genetic Rearrangements Can Modify Chromatin Features at Epialleles
Source: PLoS Genet. 2011 Oct 20;7(10):e1002331. doi: 10.1371/journal.pgen.1002331 (PMC3197671; doi:10.1371/journal.pgen.1002331)
Supplement: Table S1 — Normalization of small RNA libraries using Bowtie. (PPT) [file pgen.1002331.s008.ppt]

## Slide 1
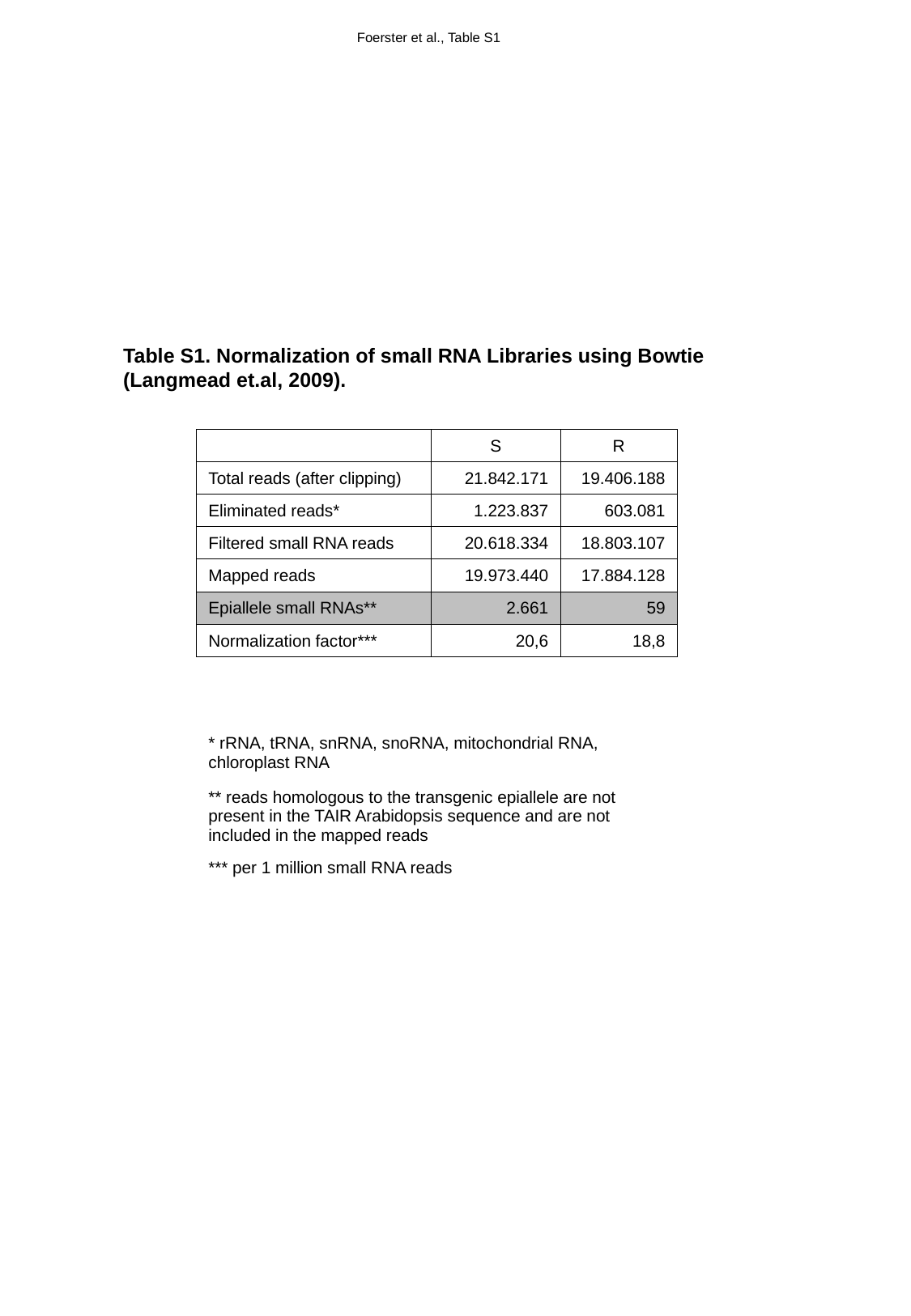

Foerster et al., Table S1
Table S1. Normalization of small RNA Libraries using Bowtie (Langmead et.al, 2009).
| | S | R |
| --- | --- | --- |
| Total reads (after clipping) | 21.842.171 | 19.406.188 |
| Eliminated reads\* | 1.223.837 | 603.081 |
| Filtered small RNA reads | 20.618.334 | 18.803.107 |
| Mapped reads | 19.973.440 | 17.884.128 |
| Epiallele small RNAs\*\* | 2.661 | 59 |
| Normalization factor\*\*\* | 20,6 | 18,8 |
| | | |
| \* rRNA, tRNA, snRNA, snoRNA, mitochondrial RNA, chloroplast RNA | | |
| \*\* reads homologous to the transgenic epiallele are not present in the TAIR Arabidopsis sequence and are not included in the mapped reads | | |
| \*\*\* per 1 million small RNA reads | | |
