# Supplementary material for: Genetic Rearrangements Can Modify Chromatin Features at Epialleles
Source: PLoS Genet. 2011 Oct 20;7(10):e1002331. doi: 10.1371/journal.pgen.1002331 (PMC3197671; doi:10.1371/journal.pgen.1002331)
Supplement: Table S3 — Summary of small RNAs reads in epialleles. (PPT) [file pgen.1002331.s010.ppt]

## Slide 1
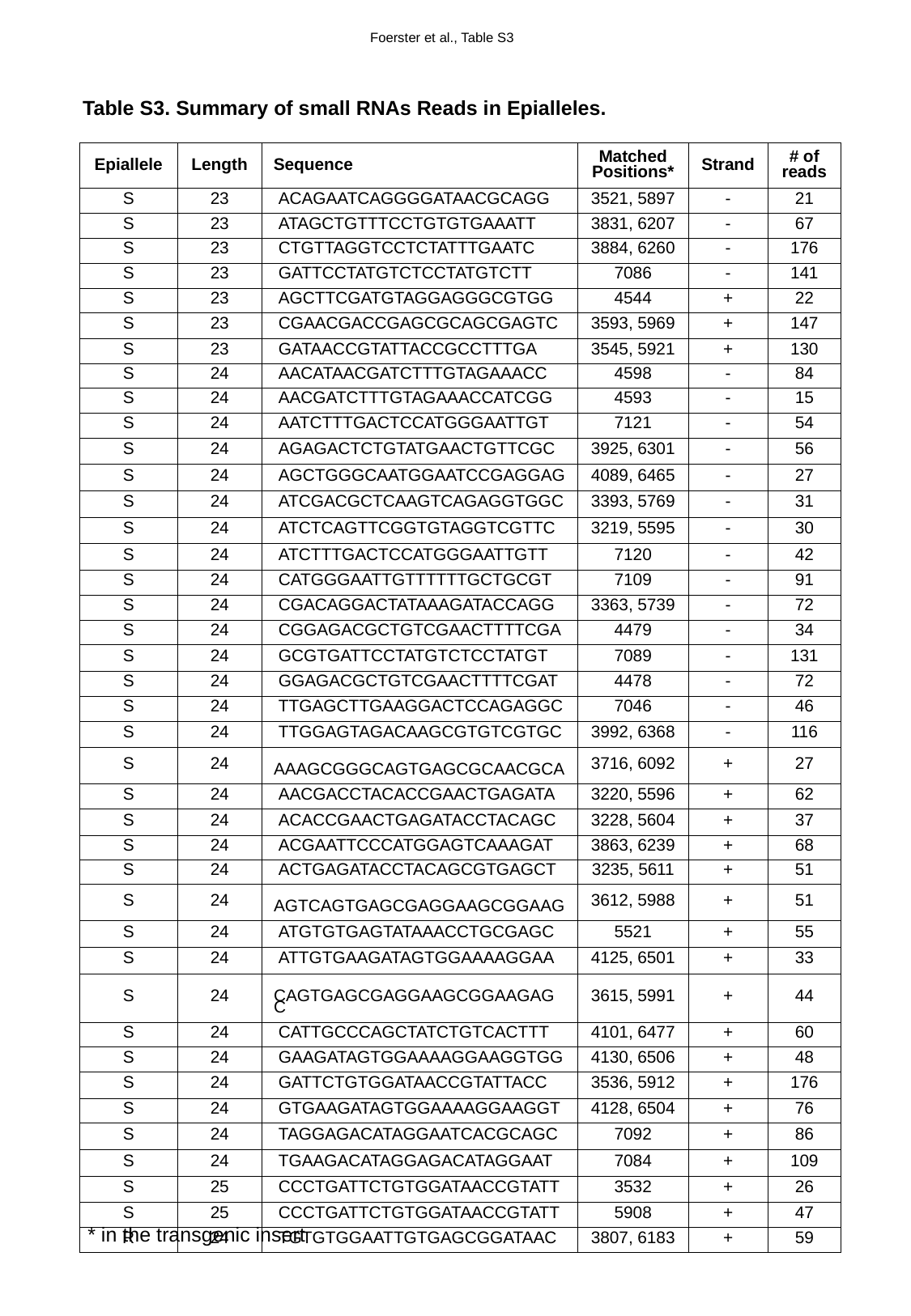

Foerster et al., Table S3
Table S3. Summary of small RNAs Reads in Epialleles.
| Epiallele | Length | Sequence | Matched Positions\* | Strand | # of reads |
| --- | --- | --- | --- | --- | --- |
| S | 23 | ACAGAATCAGGGGATAACGCAGG | 3521, 5897 | - | 21 |
| S | 23 | ATAGCTGTTTCCTGTGTGAAATT | 3831, 6207 | - | 67 |
| S | 23 | CTGTTAGGTCCTCTATTTGAATC | 3884, 6260 | - | 176 |
| S | 23 | GATTCCTATGTCTCCTATGTCTT | 7086 | - | 141 |
| S | 23 | AGCTTCGATGTAGGAGGGCGTGG | 4544 | + | 22 |
| S | 23 | CGAACGACCGAGCGCAGCGAGTC | 3593, 5969 | + | 147 |
| S | 23 | GATAACCGTATTACCGCCTTTGA | 3545, 5921 | + | 130 |
| S | 24 | AACATAACGATCTTTGTAGAAACC | 4598 | - | 84 |
| S | 24 | AACGATCTTTGTAGAAACCATCGG | 4593 | - | 15 |
| S | 24 | AATCTTTGACTCCATGGGAATTGT | 7121 | - | 54 |
| S | 24 | AGAGACTCTGTATGAACTGTTCGC | 3925, 6301 | - | 56 |
| S | 24 | AGCTGGGCAATGGAATCCGAGGAG | 4089, 6465 | - | 27 |
| S | 24 | ATCGACGCTCAAGTCAGAGGTGGC | 3393, 5769 | - | 31 |
| S | 24 | ATCTCAGTTCGGTGTAGGTCGTTC | 3219, 5595 | - | 30 |
| S | 24 | ATCTTTGACTCCATGGGAATTGTT | 7120 | - | 42 |
| S | 24 | CATGGGAATTGTTTTTTGCTGCGT | 7109 | - | 91 |
| S | 24 | CGACAGGACTATAAAGATACCAGG | 3363, 5739 | - | 72 |
| S | 24 | CGGAGACGCTGTCGAACTTTTCGA | 4479 | - | 34 |
| S | 24 | GCGTGATTCCTATGTCTCCTATGT | 7089 | - | 131 |
| S | 24 | GGAGACGCTGTCGAACTTTTCGAT | 4478 | - | 72 |
| S | 24 | TTGAGCTTGAAGGACTCCAGAGGC | 7046 | - | 46 |
| S | 24 | TTGGAGTAGACAAGCGTGTCGTGC | 3992, 6368 | - | 116 |
| S | 24 | AAAGCGGGCAGTGAGCGCAACGCA | 3716, 6092 | + | 27 |
| S | 24 | AACGACCTACACCGAACTGAGATA | 3220, 5596 | + | 62 |
| S | 24 | ACACCGAACTGAGATACCTACAGC | 3228, 5604 | + | 37 |
| S | 24 | ACGAATTCCCATGGAGTCAAAGAT | 3863, 6239 | + | 68 |
| S | 24 | ACTGAGATACCTACAGCGTGAGCT | 3235, 5611 | + | 51 |
| S | 24 | AGTCAGTGAGCGAGGAAGCGGAAG | 3612, 5988 | + | 51 |
| S | 24 | ATGTGTGAGTATAAACCTGCGAGC | 5521 | + | 55 |
| S | 24 | ATTGTGAAGATAGTGGAAAAGGAA | 4125, 6501 | + | 33 |
| S | 24 | CAGTGAGCGAGGAAGCGGAAGAGC | 3615, 5991 | + | 44 |
| S | 24 | CATTGCCCAGCTATCTGTCACTTT | 4101, 6477 | + | 60 |
| S | 24 | GAAGATAGTGGAAAAGGAAGGTGG | 4130, 6506 | + | 48 |
| S | 24 | GATTCTGTGGATAACCGTATTACC | 3536, 5912 | + | 176 |
| S | 24 | GTGAAGATAGTGGAAAAGGAAGGT | 4128, 6504 | + | 76 |
| S | 24 | TAGGAGACATAGGAATCACGCAGC | 7092 | + | 86 |
| S | 24 | TGAAGACATAGGAGACATAGGAAT | 7084 | + | 109 |
| S | 25 | CCCTGATTCTGTGGATAACCGTATT | 3532 | + | 26 |
| S | 25 | CCCTGATTCTGTGGATAACCGTATT | 5908 | + | 47 |
| R | 24 | TGTGTGGAATTGTGAGCGGATAAC | 3807, 6183 | + | 59 |
* in the transgenic insert
