# Supplementary material for: Genetic Rearrangements Can Modify Chromatin Features at Epialleles
Source: PLoS Genet. 2011 Oct 20;7(10):e1002331. doi: 10.1371/journal.pgen.1002331 (PMC3197671; doi:10.1371/journal.pgen.1002331)
Supplement: Table S4 — Primer list. (PPT) [file pgen.1002331.s011.ppt]

## Slide 1
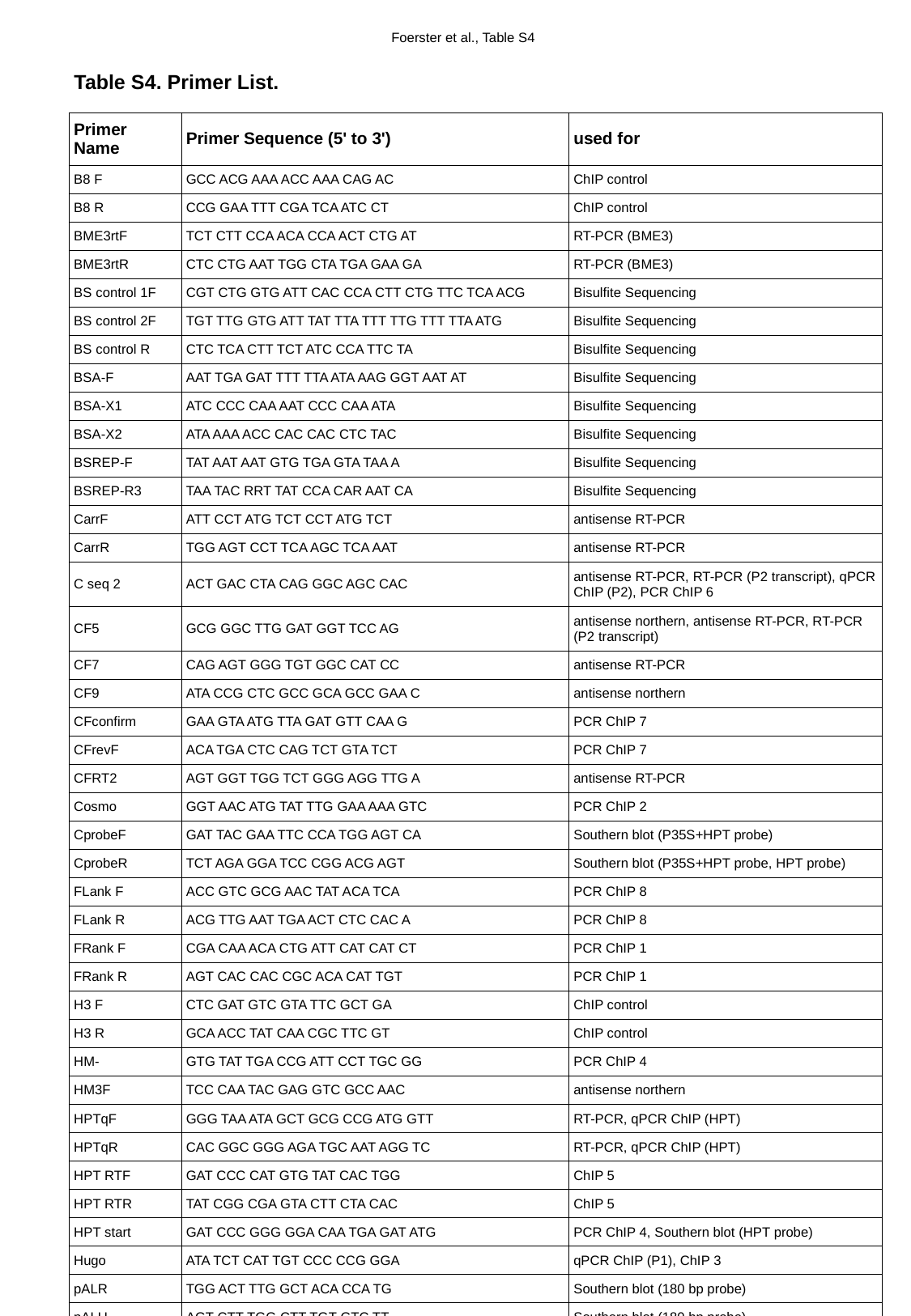

Foerster et al., Table S4
Table S4. Primer List.
| Primer Name | Primer Sequence (5' to 3') | used for |
| --- | --- | --- |
| B8 F | GCC ACG AAA ACC AAA CAG AC | ChIP control |
| B8 R | CCG GAA TTT CGA TCA ATC CT | ChIP control |
| BME3rtF | TCT CTT CCA ACA CCA ACT CTG AT | RT-PCR (BME3) |
| BME3rtR | CTC CTG AAT TGG CTA TGA GAA GA | RT-PCR (BME3) |
| BS control 1F | CGT CTG GTG ATT CAC CCA CTT CTG TTC TCA ACG | Bisulfite Sequencing |
| BS control 2F | TGT TTG GTG ATT TAT TTA TTT TTG TTT TTA ATG | Bisulfite Sequencing |
| BS control R | CTC TCA CTT TCT ATC CCA TTC TA | Bisulfite Sequencing |
| BSA-F | AAT TGA GAT TTT TTA ATA AAG GGT AAT AT | Bisulfite Sequencing |
| BSA-X1 | ATC CCC CAA AAT CCC CAA ATA | Bisulfite Sequencing |
| BSA-X2 | ATA AAA ACC CAC CAC CTC TAC | Bisulfite Sequencing |
| BSREP-F | TAT AAT AAT GTG TGA GTA TAA A | Bisulfite Sequencing |
| BSREP-R3 | TAA TAC RRT TAT CCA CAR AAT CA | Bisulfite Sequencing |
| CarrF | ATT CCT ATG TCT CCT ATG TCT | antisense RT-PCR |
| CarrR | TGG AGT CCT TCA AGC TCA AAT | antisense RT-PCR |
| C seq 2 | ACT GAC CTA CAG GGC AGC CAC | antisense RT-PCR, RT-PCR (P2 transcript), qPCR ChIP (P2), PCR ChIP 6 |
| CF5 | GCG GGC TTG GAT GGT TCC AG | antisense northern, antisense RT-PCR, RT-PCR (P2 transcript) |
| CF7 | CAG AGT GGG TGT GGC CAT CC | antisense RT-PCR |
| CF9 | ATA CCG CTC GCC GCA GCC GAA C | antisense northern |
| CFconfirm | GAA GTA ATG TTA GAT GTT CAA G | PCR ChIP 7 |
| CFrevF | ACA TGA CTC CAG TCT GTA TCT | PCR ChIP 7 |
| CFRT2 | AGT GGT TGG TCT GGG AGG TTG A | antisense RT-PCR |
| Cosmo | GGT AAC ATG TAT TTG GAA AAA GTC | PCR ChIP 2 |
| CprobeF | GAT TAC GAA TTC CCA TGG AGT CA | Southern blot (P35S+HPT probe) |
| CprobeR | TCT AGA GGA TCC CGG ACG AGT | Southern blot (P35S+HPT probe, HPT probe) |
| FLank F | ACC GTC GCG AAC TAT ACA TCA | PCR ChIP 8 |
| FLank R | ACG TTG AAT TGA ACT CTC CAC A | PCR ChIP 8 |
| FRank F | CGA CAA ACA CTG ATT CAT CAT CT | PCR ChIP 1 |
| FRank R | AGT CAC CAC CGC ACA CAT TGT | PCR ChIP 1 |
| H3 F | CTC GAT GTC GTA TTC GCT GA | ChIP control |
| H3 R | GCA ACC TAT CAA CGC TTC GT | ChIP control |
| HM- | GTG TAT TGA CCG ATT CCT TGC GG | PCR ChIP 4 |
| HM3F | TCC CAA TAC GAG GTC GCC AAC | antisense northern |
| HPTqF | GGG TAA ATA GCT GCG CCG ATG GTT | RT-PCR, qPCR ChIP (HPT) |
| HPTqR | CAC GGC GGG AGA TGC AAT AGG TC | RT-PCR, qPCR ChIP (HPT) |
| HPT RTF | GAT CCC CAT GTG TAT CAC TGG | ChIP 5 |
| HPT RTR | TAT CGG CGA GTA CTT CTA CAC | ChIP 5 |
| HPT start | GAT CCC GGG GGA CAA TGA GAT ATG | PCR ChIP 4, Southern blot (HPT probe) |
| Hugo | ATA TCT CAT TGT CCC CCG GGA | qPCR ChIP (P1), ChIP 3 |
| pALR | TGG ACT TTG GCT ACA CCA TG | Southern blot (180 bp probe) |
| pALU | AGT CTT TGG CTT TGT GTC TT | Southern blot (180 bp probe) |
| P35SF | CAG TCT CAG AAG ACC AAA GGG | ChIP 3, ChIP 6 |
| P35S-F | GTG ATA TCT CCA CTG ACG TAA GGG | antisense northern, antisense RT-PCR, qPCR ChIP (P1/P2), RT-PCR (P2) |
| Politan | ATA ATG GGA AGG TGA AAT GGC A | PCR ChIP 2 |
| siRNA02 | GTT GAC CAG TCC GCC AGC CGA T | small RNA northern |
| siRNA1003 | ATG CCA AGT TTG GCC TCA CGG TCT | small RNA northern |
| U6 probe | TCA TCC TTG CGC AGG GGC CA | Poly(A) northern |
