# Supplementary material for: Genetic Rearrangements Can Modify Chromatin Features at Epialleles
Source: PLoS Genet. 2011 Oct 20;7(10):e1002331. doi: 10.1371/journal.pgen.1002331 (PMC3197671; doi:10.1371/journal.pgen.1002331)
Supplement: Text S1 — Supplemental methods and references. (DOC) [file pgen.1002331.s012.doc]

**Text S1: Supplemental methods and references**

**Callus Induction**

Callus samples were obtained by cutting cotyledons from 2 week-old seedlings grown on standard GM plates. Explants were transferred onto GM callus-inducing medium, consisting of GM base supplemented with auxin (α-naphtalene acetic acid, NAA, 2 μg/ml and 2,4-dichlorophenoxyacetic acid, 2,4-D, 0.05 μg/ml), cytokinin (6-benzyl aminopurine, BAP, 0.15 μg/ml and kinetin, 0.15 μg/ml), 3% (w/v) sucrose and 4% (w/v) glucose.

**Transient Transformation in Cell Suspension Culture**

Cell suspension culture was initiated from about 500 sterile seeds (Col) germinated in liquid Murashige-Skoog medium supplemented with 3% (w/v) sucrose, 2 x Gamborg B5 vitamins (Duchefa), 0.5 μg/ml 2,4-dichloro-phenoxyacetic acid, 2 μg/ml 6-(γ,γ-methylallylamino)-purine riboside. Suspension culture was propagated every week by pipetting 5 ml of suspension culture into a new sterile flask containing 20 ml fresh medium. Cultures were kept at 25°C, shaking at 130 rpm in the dark.

The non-coding sequence downstream of P2 in the epiallele was cloned in both orientations into plasmid pCBK04, replacing the 35S promoter via *BamHI* and *PstI* restriction sites. The constructs were transformed into electrocompetent cells of *Agrobacterium tumefaciens* strain (AGL). *Agrobacterium* cultures were initiated by inoculation of 25 ml of LB medium supplemented with Rifampicin (40 μg/ml), Carbenicillin (100 μg/ml) and Kanamycin (50 μg/ml) to select for cells containing the plasmids. The cultures were grown to an OD_600_ of 0.9 to 1.0, collected by centrifugation and resuspended in 1/10th of the original volume in cell suspension medium. Cell suspension was treated with 0.5 mM acetosyringone prior to inoculation with Agrobacteria to facilitate the transformation procedure. Cell suspension aliquots were inoculated with Agrobacterium cultures containing the respective plasmids and co-cultivated for 3 days at 25°C shaking in the dark at 130 rpm. Subsequently, the aliquots were washed several times with suspension medium to remove bacteria, followed by fixation and GUS staining.

***In situ* GUS Detection**

GUS activity was detected by staining plant tissue or cells in 0.1 M sodium phosphate buffer pH 7.0, 10 mM EDTA, 0.1% Triton X-100, 100 μg/ml chloramphenicol, 2 mM potassium ferrocyanide, 2 mM potassium ferricyanide and 0.5 mg/ml X-glucuronide after 30 min vacuum infiltration and overnight incubation at 37°C. Subsequent washes with 70% ethanol at 37°C removed chlorophyll and enhanced the contrast. All samples were analysed using a Leica MZ16FA binocular microscope with a Leica DFC300FX CCD camera. Images were acquired with the Leica Application Suite and processed with Adobe Photoshop (Adobe).

**Poly(A) RNA Enrichment**

MicroPoly(A)Purist Kit (Ambion) was used to isolate poly(A) RNA from total RNA obtained with Trizol (Invitrogen) RNA isolation.

**Immunolocalisation**

Young rosette leaves were fixed under a vacuum for 20 minutes in 4% formaldehyde in TRIS buffer (10 mM Tris-HCl, 10 mM Na_2_ EDTA, 100 mM NaCl, and 0.1% Triton X-100, pH 7.5). Nuclei were isolated by chopping the leaves in LB01 buffer and stained with 4',6-diamidino-2-phenylindole (1 μg/ml). 2C nuclei were sorted in a FACSAria flow cytometer (Becton Dickinson). Approximately 2000 nuclei were applied on microscope slides in a drop of buffer (100 mM Tris, 50 mM KCl, 2 mM MgCl_2_, 0.05% Tween, 5% sucrose), air-dried and used for FISH or stored at −20°C until use. The following BAC clones were prepared as probes: F13M14, T7M13, T14E10, T5N23, T9N14, and T10D10. BAC DNA was labeled by nick translation using biotin-dUTP or digoxigenin-dUTP . The 180 bp repeat was labelled by PCR from the pAL1 plasmid using Cy3-dUTP (Amersham) and the primers AGTCTTTGGCTTTGTGTCTT and TGGACTTTGGCTACACCATG. Pepsin treatment of nuclei prior to FISH was performed as described . Labeled BACs were precipitated and resuspended in 20 μl hybridization buffer (50% formamide, 10% dextran sulphate, 2x SSC, 50 mM sodium phosphate, pH 7.0) per slide. After mounting the probe, the slides were placed on a heat block at 80°C for 2 minutes and then incubated in a moist chamber at 37°C for 12-16 hours. Post-hybridization washes and detection steps were conducted as described . Biotin-dUTP was detected by goat anti-avidin conjugated with biotin (1:200; Vector Laboratories) and avidin conjugated with Texas Red (1:1000; Vector Laboratories), digoxigenin-dUTP by mouse anti-digoxigenin (1:250; Roche) and goat anti-mouse conjugated with Alexa-488 (1:200; Molecular Probes). Cy3-dUTP labeling was evaluated directly. Nuclei and chromosomes were counterstained with DAPI (1 μg/ml) in Vectashield (Vector Laboratories). Microscopic evaluation and image analysis was as described .

**Quantification of Global DNA Methylation**

Total cytosine methylation was determined by cation exchange high pressure liquid chromatography as described by . All samples were analysed in triplicate and 5-^m^dC values were expressed as a percentage of total cytosine.

**Supplemental References**

1. Dolezel J, Binarova P, Lucretti S (1989) Analysis of nuclear DNA content in plant cells by flow cytometry. Biologia Plantarum 31: 113-120.

2. Ward P (2002) FISH probes and labelling techiques. In: Beatty B, Mai S, Squire J, editors. FISH. Oxford: Oxford University Press. pp. 5-28.

3. Henegariu O, Bray-Ward P, Ward DC (2000) Custom fluorescent-nucleotide synthesis as an alternative method for nucleic acid labeling. Nat Biotechnol 18: 345-348.

4. Probst AV, Fransz PF, Paszkowski J, Mittelsten Scheid O (2003) Two means of transcriptional reactivation within heterochromatin. Plant J 33: 743-749.

5. Martinez-Zapater J, Estelle A, Somerville R (1986) A highly repeated sequence in *Arabidopsis thaliana*. Mol Gen Genet 204: 417-423.

6. Pecinka A, Schubert V, Meister A, Kreth G, Klatte M, et al. (2004) Chromosome territory arrangement and homologous pairing in nuclei of Arabidopsis thaliana are predominantly random except for NOR-bearing chromosomes. Chromosoma 113: 258-269.

7. Schubert I, Fransz PF, Fuchs J, de Jong JH (2001) Chromosome painting in plants. Methods Cell Sci 23: 57-69.

8. Pecinka A, Kato N, Meister A, Probst AV, Schubert I, et al. (2005) Tandem repetitive transgenes and fluorescent chromatin tags alter local interphase chromosome arrangement in Arabidopsis thaliana. J Cell Sci 118: 3751-3758.

9. Rozhon W, Baubec T, Mayerhofer J, Mittelsten Scheid O, Jonak C (2008) Rapid quantification of global DNA methylation by isocratic cation exchange high-performance liquid chromatography. Anal Biochem 375: 354-360.
